# Assessing the reproductive biology of the Greenland shark (*Somniosus microcephalus*)

**DOI:** 10.1371/journal.pone.0238986

**Published:** 2020-10-07

**Authors:** Julius Nielsen, Rasmus B. Hedeholm, Arve Lynghammar, Leon M. McClusky, Bjørn Berland, John F. Steffensen, Jørgen S. Christiansen

**Affiliations:** 1 Greenland Institute of Natural Resources, Nuuk, Greenland; 2 Faculty of Biosciences, Fisheries and Economics, UiT The Arctic University of Norway, Tromsø, Norway; 3 Faculty of Health Sciences, UiT The Arctic University of Norway, Narvik, Norway; 4 University of Bergen, Bergen, Norway; 5 Department of Biology, University of Copenhagen, Helsingør, Denmark; 6 Environmental and Marine Biology, Åbo Akademi University, Turku, Finland; University of Adelaide, AUSTRALIA

## Abstract

The Greenland shark (*Somniosus microcephalus*, Squaliformes: Somniosidae) is a long-lived Arctic top predator, which in combination with the high historical and modern fishing pressures, has made it subject to increased scientific focus in recent years. Key aspects of reproduction are not well known as exemplified by sparse and contradictory information e.g. on birth size and number of pups per pregnancy. This study represents the first comprehensive work on Greenland shark reproductive biology based on data from 312 specimens collected over the past 60 years. We provide guidelines quantifying reproductive parameters to assess specific maturation stages, as well as calculate body length-at-maturity (TL_50_) which was 2.84±0.06 m for males and 4.19±0.04 m for females. From the available information on the ovarian fecundity of Greenland sharks as well as a meta-analysis of Squaliform reproductive parameters, we estimate up to 200–324 pups per pregnancy (depending on maternal size) with a body length-at-birth of 35–45 cm. These estimates remain to be verified by future observations from gravid Greenland sharks.

## Introduction

The Greenland shark (*Somniosus microcephalus*, Squaliformes: Somniosidae) is a long-lived and migratory top predator [1–3]. Considering the high historical and modern fishing pressures on Greenland sharks [4, 5], pertinent questions arise concerning population size and proper conservation actions across its main distribution area throughout the Arctic [6]. The fundamental biology of the Greenland shark is therefore crucial to understand, yet many aspects are not well known including the reproductive biology [5, 7].

When evaluating the reproductive biology of live-bearing sharks, and in particular the reproductive output of a Squaliform shark, it is important to differentiate between ovarian fecundity and uterine fecundity. Ovarian fecundity refers to the number of unfertilized, ripe ova in the ovaries, whereas uterine fecundity is the number of fertilized ova/developing pups in the uterus. Beside the study by Yano et al. [8], where body length-at-maturity was estimated to 3 m in total length (TL) for males and above 4 m for females, few and somewhat conflicting observations—especially on fecundity and birth size—have been published for Greenland sharks. The earliest information on their ovarian fecundity is from Lütken [9] mentioning as much as two barrels of ova the size of small goose-eggs in large females–a trait that almost 40 years later was quantified for a ~4 m female carrying ~500 soft ova, each ‘the size of a hen’s egg’ [10]). A high ovarian fecundity of Greenland sharks was also noted by Nikolsky [11] with 500 ova up to 8.0 cm in diameter and by Nielsen et al. [12] reporting 455 ovarian ova with a mean diameter of 5.1 cm from a female of 4.5 m TL. Although MacNeil et al. [7] mention “Numerous *S*. *microcephalus* with fertilized eggs in utero, including a female from Cumberland Sound that contained c. 1800 fertilized eggs” (i.e. a gravid female), these observations have been disconfirmed by the authors (personal communication with M. A. MacNeil and A. T. Fisk). Therefore, the only validated observation of a gravid Greenland shark is Koefoed [13], who reported 10 near-term and similarly sized fetuses of 37 cm TL from the right uterus of a ~5 m female (see Discussion). That find established that the Greenland shark is aplacental viviparous and not egg-laying, which at the time had been debated for nearly a century [10, 14]. Koefoed’s report also raised questions concerning the species’ actual uterine fecundity, which seemed low as also supported by Bjerkan’s report of a gravid female with a single fetus of ~98 cm [14]. Based on these two reports, the uterine fecundity of Greenland sharks has been considered relatively low (1–10 pups per pregnancy) and the birth size range to be large (40–100 cm TL, [7]). We believe however that these numbers should be revised based on the findings of this study.

Birth size and fecundity vary greatly among Chondrichthyes. Some species give birth to few relatively large pups (e.g. *Carcharodon carcharias*, Lamniformes, with ten pups >1 m TL [15]), while others give birth to hundreds of relatively small pups (e.g. *Rhincodon typus*, Orectolobiformes, with 300 pups of 58–64 cm TL [16]). For viviparous sharks, this variation is associated with different reproductive modes and the degree of maternal supply of nutrition during gestation. Species with nutrition derived solely from yolk are lecitotrophic, whereas matrotrophic species provide additional nutrition during embryonic development. The degree of maternal supplement follows a continuum across species from none to extensive [17, 18]. Matrotrophic reproductive modes can be further classified as histotrophy (uterine secretions of either mucoid or lipids), oophagy (feeding on ova), adelphophagy (a derivate of oophagy, where siblings are being cannibalized) and placentotrophy (nutritional transfer via placenta-like organ) [18]. An overall phylogenetic pattern is evident and closely related sharks often exhibit similar reproductive strategies [18, 19]. Furthermore, although it has been debated how to discern correctly between lecitotrophic viviparity and limited histotrophy [20], it is commonly accepted that these two strategies (where the latter is the natural progression of the former), are the only reproductive strategies within the relatively well investigated Squaliform order [17, 21–23].

Here, we address the reproductive biology of the Greenland shark with three specific aims. Aim 1 describes and quantifies sex-specific maturation stages as defined for aplacental viviparous sharks. Aim 2 establishes body length-at-maturity for males and females. Aim 3 deduces the uterine fecundity (i.e. number of pups per pregnancy), birth size and elucidates aspects of the reproductive cycle of the Greenland shark. In the absence of gravid females, this latter aim is addressed using data of mature Greenland shark females and by a meta-analysis on selected reproductive parameters of other Squaliforms. All aims combined, our study provides the first comprehensive insight into the reproductive biology of the Greenland shark, which is assigned as ‘Near Threatened’ by the International Union for Conservation of Nature (IUCN) Red List of Threatened Species [24].

## Materials and methods

### Data sets

**Fig 1:**
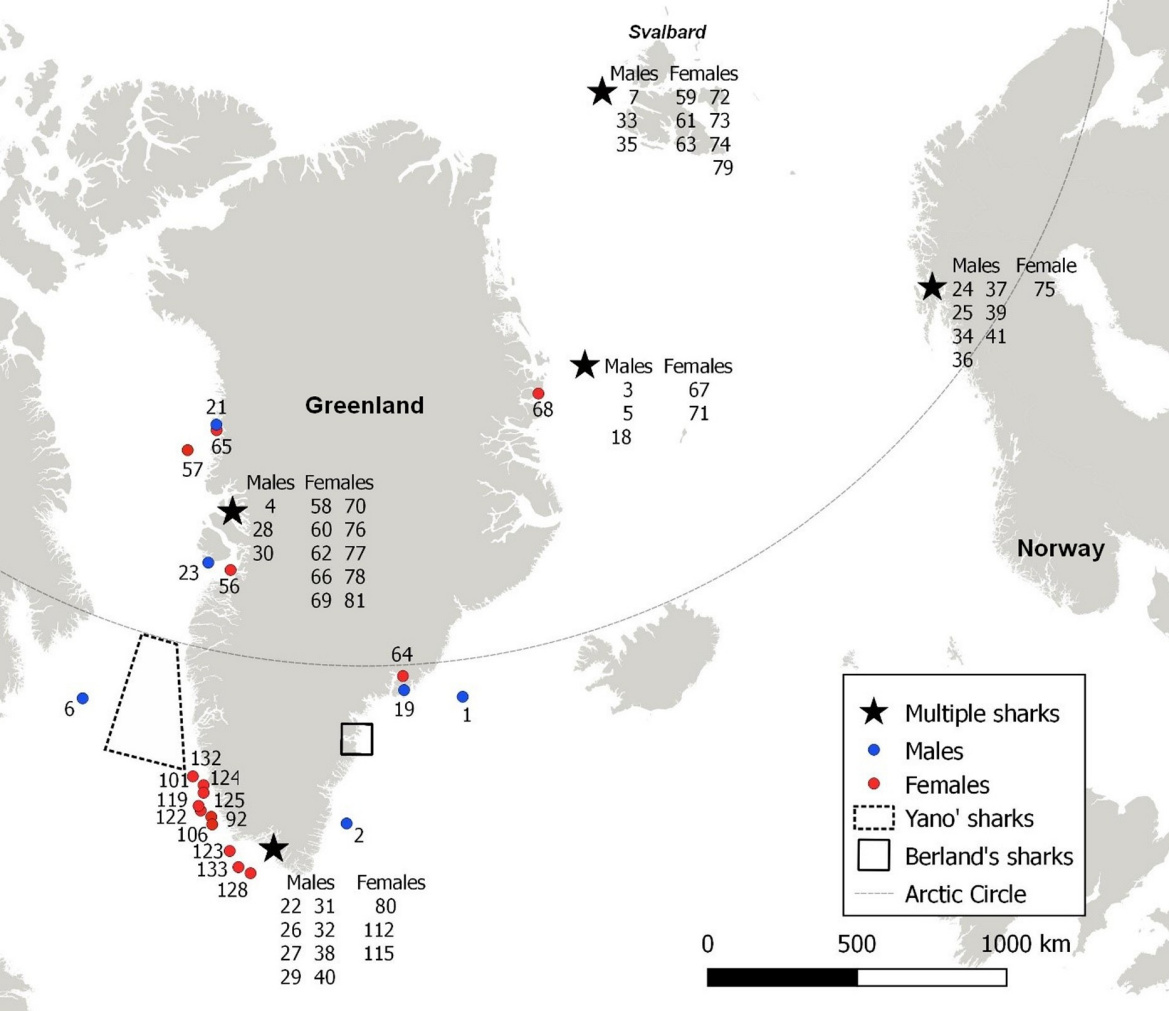
Capture locations of sharks. Capture locations of single sharks (dots) or multiple sharks (stars) caught by JN (blue: male; red: female). For each mark, the associated shark identification number(s) are listed (see S1 and S2 Tables). The sharks of Berland (N = 207) and Yano (N = 36) were all caught within relatively small geographical areas indicated by solid and punctuated line, respectively. For these, shark identification numbers are not shown.

The analyzed Greenland shark data are from three different sources (Table 1). The first source consists of unpublished data collected by the late professor emeritus Bjørn Berland, who participated in targeted fisheries with the Norwegian sealing vessels Brandal in 1959 and Polaric in 1960. During these expeditions, Berland gathered information on the reproductive biology from 207 sharks sampled in Umivik Bay and nearby offshore banks in Southeast Greenland (Fig 1). The second source consists of published reproductive data from 36 sharks collected by Kazunari Yano [8] in offshore waters of Southwest Greenland (Fig 1) between 1987 and 1991. The third and most comprehensive source, in terms of data from individual sharks, comprises original data from 69 sharks collected by Julius Nielsen (JN) during scientific expeditions in Greenland and Norway between 2012 and 2019 (Fig 1). These sharks were mainly obtained as bycatch during annual fish surveys conducted by the Greenland Institute of Natural Resources in inshore and offshore waters of West and East Greenland with RVs Pâmiut and Sanna. Some of JN’s sharks were also obtained from Greenland and Norwegian waters (including Svalbard) via multiple expeditions using longlines and fishing rods from RVs Dana, Porsild, Helmer Hanssen and Johan Ruud, and the facilities of Andørja Adventures in northern Norway. The sampling by JN was conducted under the auspices of the ‘Old & Cold—Greenland shark project’ at the University of Copenhagen (http://bioold.science.ku.dk/jfsteffensen/OldAndCold/), and was carried out in accordance with laws, regulations and authorization of the Government of Greenland (Ministry of Fisheries, Hunting & Agriculture, document numbers 565466, 935119, 20179208, C-17-129, C-15-17, and C-13-16) and the Norwegian Food Safety Authority (ID: 8727. Case: 16/150243).

**Table 1:** Overview of Greenland shark data sources.

Source	Sampling period	Aim 1		Aim 2	
		N_males_	N_females_	N_males_	N_females_
Berland	1959–1960	2	39	13	194
Yano	1987–1991	12	0	12	24
Nielsen	2012–2019	30	39	30	39
*Total*		*44*	*78*	*55*	*257*

Sources, sampling periods and numbers of sharks for Aims 1 and 2 (cf. S1–S3 Tables).

Because data for this study have very diverse origins, the amount of information from each specimen varies within and among data sources. Each data source and the corresponding number of specimens available for Aim 1 (Maturation stages) and Aim 2 (Body length-at-maturity) are shown in Table 1. All body lengths (TL) are rounded off to nearest 10 cm bin for subsequent comparative analyses. Calculations and statistics were made in R [25].

### Aim 1: Maturation stages

Greenland sharks were categorized according to a predefined general maturation scale for aplacental viviparous sharks. This maturation progression was originally developed by Stehmann [26] and encompasses three stages for males (Stage 1 ‘Immature’, Stage 2 ‘Maturing’, and Stage 3 ‘Mature’) and seven stages for females (Stage 1 ‘Immature’, Stage 2 ‘Developing’, Stage 3 ‘Ripening’, Stage 4 ‘Early Gravid’, Stage 5 ‘Midterm Gravid’, Stage 6 ‘Late Gravid’, and Stage 7 ‘Post-natal’. An additional stage, Stage X(7) was added for females which had given birth previously and returned to a previous stage (which the X refers to) in the maturation progression (i.e. Stage 2 or 3).

Data are available for 44 males that could be categorized based on both external and internal parameters. Internal parameters are testes length (N = 12), testes mass (N = 29), testes form (N = 13), testes colour (N = 15) and the presence of sperm in the seminal vesicle (N = 5). External parameters encompass claspers length (from the rear end of cloaca to the tip of clasper, N = 28), rigidity of claspers (soft or calcified, N = 36) and if the tip of claspers extends beyond the tip of pelvic fins (N = 28).Data are available from 78 females and encompass only internal parameters. These are ovary length (N = 50), ovary mass (N = 35), ovary colour (N = 35), maximum diameter of ova (N = 42), number of ripe/ripening ova (N = 3), uterus size (small or enlarged, N = 10), and the presence of villi lining the inner surface of the uterus (N = 47).

### Aim 2: Body length-at-maturity

To evaluate body length-at-maturity, sharks were categorized as either ‘immature’ or ‘mature’. Data encompass all sharks of Aim 1 (44 males and 78 females) for which Stage 1 males (N = 17) and Stage 1 females (N = 26) were categorized as ‘immature’. Stages 2–3 males (N = 27) and Stages 2–7 females (N = 51), were categorized ‘mature’. Such maturity assignment is similar to length-at-maturity studies for other Squaliforms (see [27–29]). Additional data from Berland’s original material comprises testes length from 11 males of 1.5–3.4 m TL (nos. 45–55, S1 Table). Although testes length is not a strong parameter to differentiate between maturation stages, these males were categorized as either ‘immature’ or ‘mature’ based on the findings of Aim 1. Berland’s material also included body length information from 155 females of 3.4–4.9 m TL (nos. 158–312, S3 Table) that originally were assigned on sight to one of only three maturation stages: 1) Immature females with small-sized ova and ovaries, and insignificant oviducts without posterior swelling; 2) Maturing females with ova and ovaries of increasing size, and oviducts with dilated posterior part, yet internal surface of the uteri without villi; 3) Mature females with ova and ovaries of either small or large size (and sometimes degenerated), as well as greatly enlarged oviducts in the posterior half (that is the uteri), which also has an internal surface lined with numerous villi. This categorization (‘Immature’, ‘Maturing’, or ‘Mature’) corresponds either to Stage 1, Stage 2 or Stage 7 and X(7) of Aim 1 and therefore, these 155 on-sight evaluated females, could precisely be categorized as either ‘immature’ or ‘mature’. Lastly, Yano et al. [8] report 24 females of 0.7–4.8 m TL (nos. 134–157, S2 Table) from which ovary mass was available allowing these to be categorized as ‘immature’ or ‘mature’ based on the findings of Aim 1. The total dataset for Aim 2 was thus 44+11 = 55 males and 78+155+24 = 257 females.

Maturity ogives were fitted to the data by a generalized linear model (GLM), assuming a binomial error distribution and logit link function. The body length-at-maturity (TL_50_) for males and females respectively was calculated and visualized with 95% confidence limits.

### Aim 3: Fecundity, body length-at-birth, and the reproductive cycle

To assess uterine fecundity, body length-at-birth and aspects of the reproductive cycle of Greenland sharks, two kinds of data were analyzed in concert: 1) Original data on Greenland shark ovarian fecundity and liver mass, and 2) data of female reproductive parameters from other Squaliforms acquired from the scientific literature.

Greenland shark: Data on ovarian fecundity were available from four females (one in Stage 2 and three in Stage 3) whereas data on liver mass were available from 14 mature females. Additional data on liver masses from immature females (N = 21) and immature/mature males (N = 16) are included for comparison between sex and maturation stages. Neither hepatosomatic nor gonadosomatic indices could be calculated as body weight was not recorded for most of the collected specimens.

Other Squaliforms: Data from 23 species were analyzed to identify trends of ovarian fecundity, uterine fecundity, size of ripe ova, body length-at-birth and/or reproductive strategy across the order (S4 Table). These species represent all seven families of Squaliforms as follows: Centrophoridae/Gulper sharks, N = 8 [21, 23, 29–37], Dalatiidae/Kitefin sharks, N = 1 [38–40], Echinorhinidae/Bramble sharks, N = 1 [41, 42], Oxynotidae/Rough sharks, N = 2 [43–46], Etmopteridae/Lantern sharks, N = 4 [23, 27, 47–50], Somniosidae/Sleeper sharks, N = 4 [22, 30, 31, 33, 35, 51–53], J. Guallart, unpublished data), and Squalidae/Dogfish sharks, N = 3 [54–64]. Although the extent of data sets varies between species and studies, we contend that such phylogenetic comparison allows for identifying general reproductive patterns among Squaliforms including Somniosidae to which the Greenland shark belongs.

Ovarian and uterine fecundity often reflect each other within Squaliforms [47] and we suggest that the potential number of fetuses (i.e. number of pups per pregnancy) can be deduced from ovarian observations. To do this, we calculated the highest number of near-term fetuses (U) over the highest number of ripe ova (O). This species-specific ratio is referred to as the UO_max_-ratio (%) and reflects the maximum capacity of the uterus based on number of ripe ova in the ovaries. In case of multiple studies reporting both ovarian and uterine fecundities for a given species, the study with the largest female sample size was used.

## Results

### Aim 1: Maturation stages

**Fig 2:**
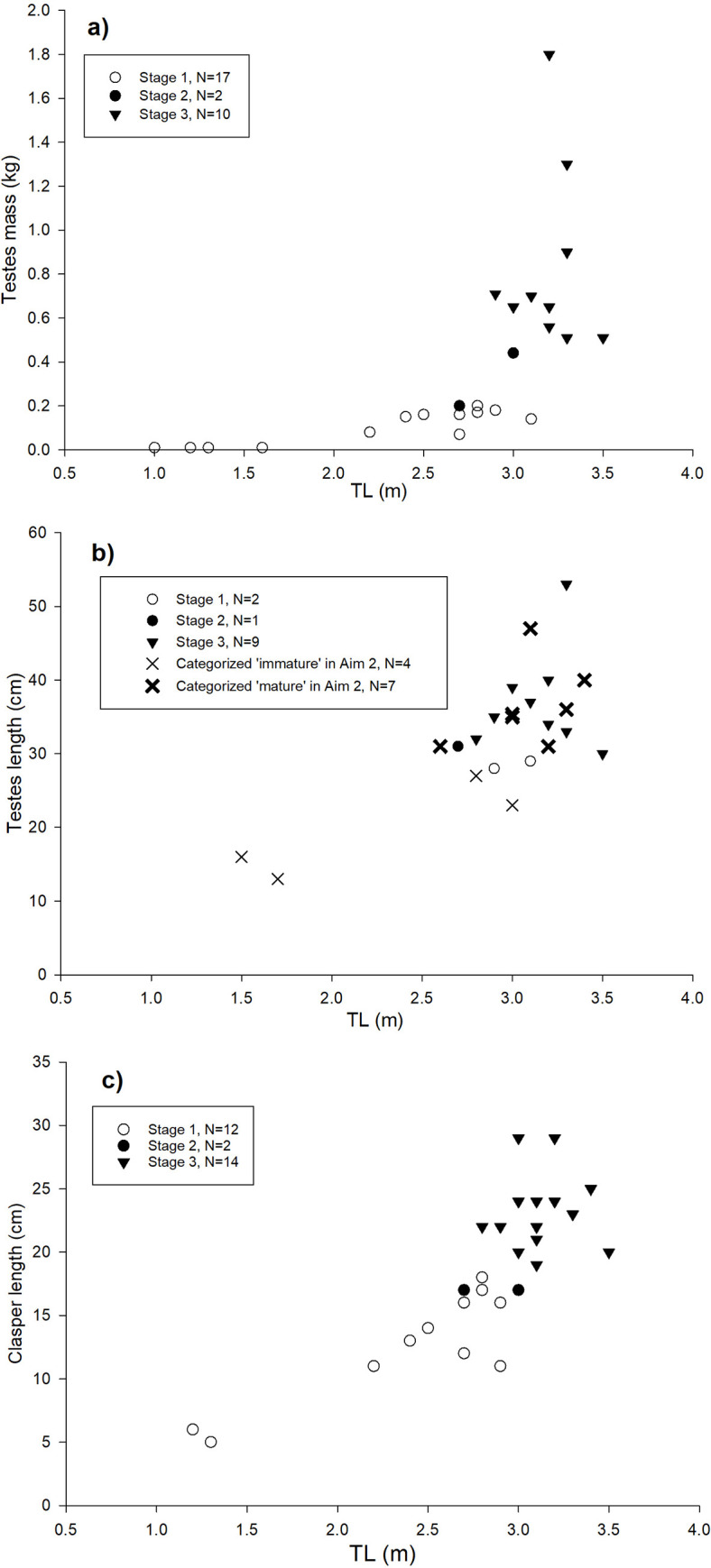
Reproductive parameters for males. (a) testes mass, (b) testes length, (c) clasper length, against TL and maturation stages.

Data on reproductive parameters obtained for males (N = 44, 1.0–3.8 m TL, nos. 1–44) and females (N = 78, 1.5–4.9 m TL, nos. 56–133) are presented in S1 and S2 Tables, respectively. The variation in testes mass, testes length and clasper length across TL, and between maturation stages, is shown in Fig 2A to 2C. The categorization of 44 males was: 17 in Stage 1 (1.0–3.1 m TL, nos. 1–17) having poorly developed reproductive organs (S1A and S1B Fig), three in Stage 2 (2.7–3.0 m TL, nos. 18–20) with extended but non-calcified claspers, and 24 in Stage 3 (2.8–3.8 m TL, nos. 21–44) with enlarged reproductive organs (S1C and S1D Fig). Six of the Stage 3 males had claspers with an extruded spur at capture (S1E and S1F Fig) of which three emitted sperm from the urogenital papilla upon exertion of mechanical pressure on the seminal gland. These three males were further categorized as ‘Active’ (nos. 22, 27 and 32). The most distinct parameters between male maturation stages were clasper length, clasper rigidity and testes mass which are quantified for each stage in Table 2.

**Table 2:** Maturation stages.

****Males****		
*Stage 1—Immature*	*Stage 2—Maturing*	*Stage 3—Mature*
Testes are small with a mass <0.2 kg and lengths <29 cm. Claspers are not calcified but soft, and shorter than the extreme tip of pelvic fin and measure <19 cm in length. See S1A and S1B Fig.	Enlarged testes with a mass between 0.2–0.4 kg. Claspers still soft but extend longer than pelvic fins. See S1D Fig.	Enlarged and heavily indented testes with a mass >0.5 kg and lengths >30 cm. Calcified claspers measure >19 cm. Clasper spur may be visible. Specimens with freely flowing sperm can be categorized as ‘active’. See S1c to S1F Fig.
		
****Females****		
*Stage 1—Immature*	*Stage 2—Developing*	*Stage 3—Ripening*
Oviducts and uteri are small/poorly developed. The uteri can be difficult to identify. Ovaries most often appear white and poorly developed with a mass <1 kg and lengths ≤62 cm. See S2 Fig. Ova were not developed or appeared small and granulated/grain-like. Such ova from a single specimen had a maximum diameter of 0.2 cm.	Ovaries are enlarged with a mass ≥2 kg and typically lengths >65 cm. Ova were differentiated and had a maximum diameter (in individual animals) >1 cm and <5 cm. Uteri and oviducts become more developed compared to previous stage 1. Liver mass may vary hundreds of kg for same sized sharks. For a single specimen with ova size up to 3.5 cm in diameter, the uterus was enlarged but contained no internal villi. See S3 Fig.	Very large ovaries with >400 large yolky ova of almost similar size, ≥5.0 cm in diameter (up to 8.0 cm). Numerous small ova <1.5 cm. Uteri and oviducts presumably similar to previous stage. See Fig 4.
*Stage 4—Early gravid (no data)*	*Stage 5—Midterm gravid (no data)*	*Stage 6—Late gravid (no data)*
Uteri filled with yellow segmented fertilized ova (uteri in this stage is also referred to as candle). Oviducts are enlarged and will be in all following stages.	Underdeveloped embryos in uteri having external yolk sack.	Fully formed near-term embryos in uteri with absorbed external yolk sack.
*Stage 7—Post-natal*	*Stage X (7)–Developing/ripening that has given birth previously*	
Non-gravid with exhausted brownish and flaccid ovaries. The mass from the only specimen was 4.2 kg. Ovaries are empty with only small ova or single degenerated ripe ova present. Uteri remain enlarged in this and subsequent stages with villi present on the inner epithelium. See S4A and S4B Fig.	Enlarged uteri with villi lining the inner epithelium. Ovaries are either developing (Stage 2) or ripening (Stage 3). See S5A and S5B Fig.	

Parameters for the maturation stages among the analyzed Greenland sharks. Note there are no available data for gravid stages (Stage 4–6).

**Fig 3:**
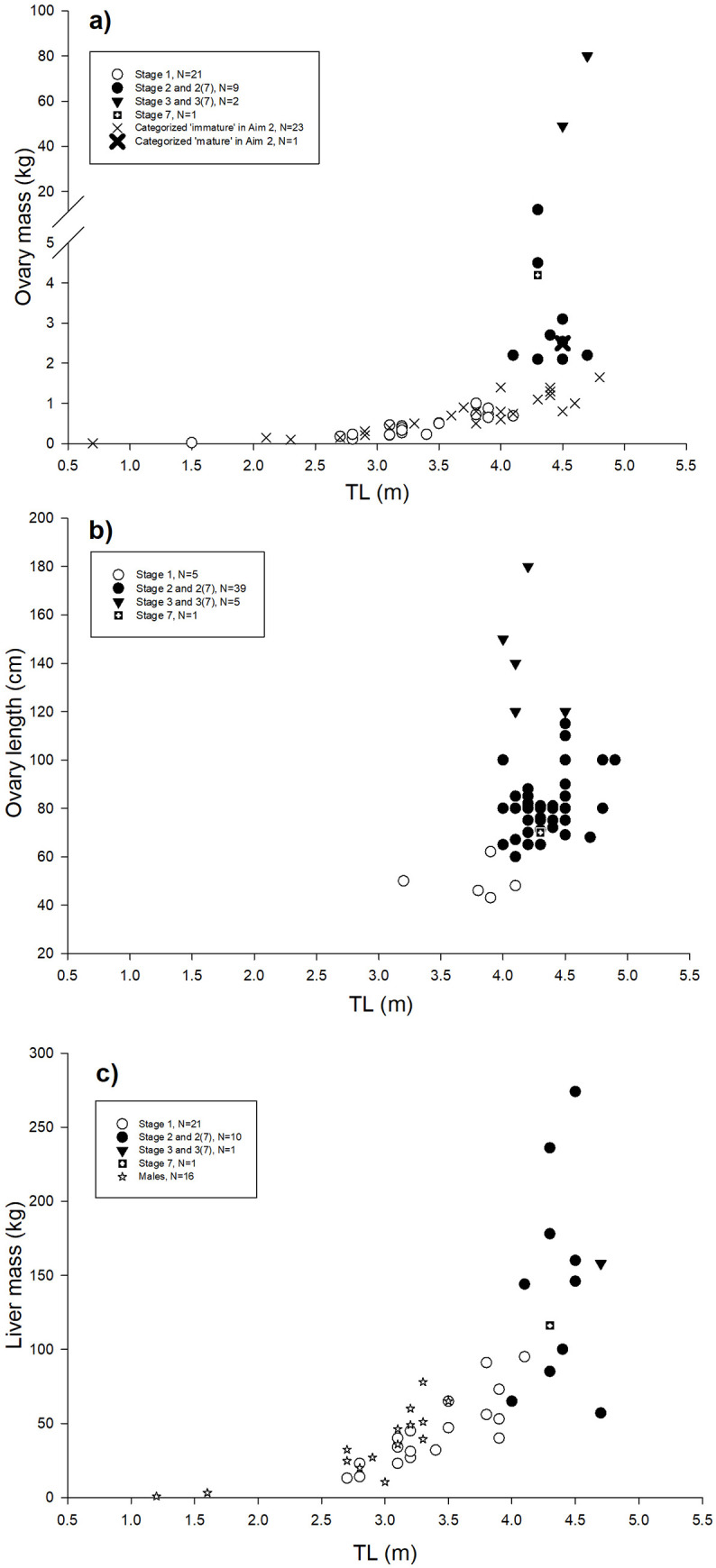
Reproductive parameters for females. (a) ovary mass, (b) ovary length, and (c) liver mass against TL and maturation stage. Note the break on y-axis in panel a and the inclusion of male liver mass in panel c.

**Fig 4:**
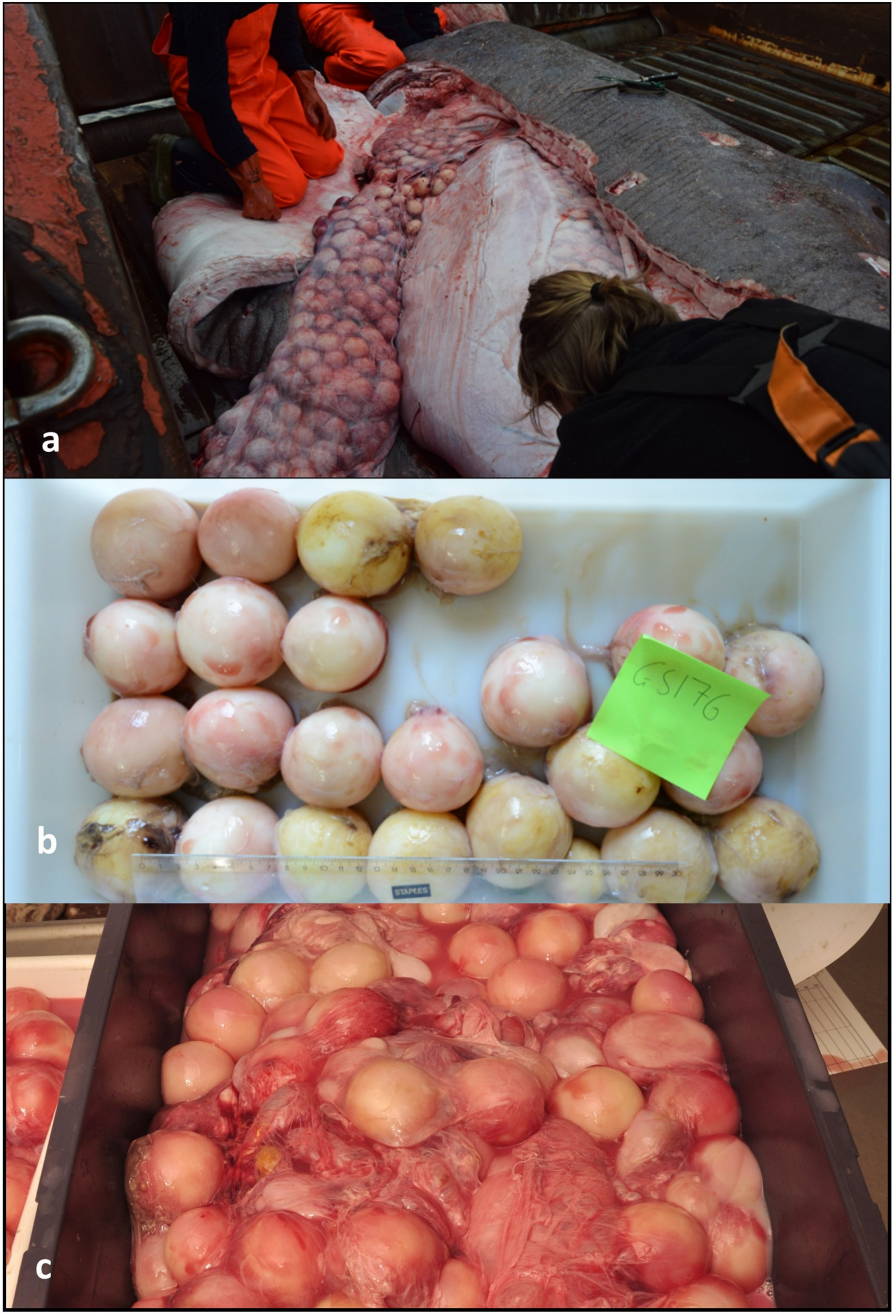
Ripening ova. (a+b) similar sized ripe/ripening ova up to 6.0 cm in diameter of Shark no. 128. (c) ripe/ripening ova up to 7.0 cm in diameter of Shark no. 132 as well as a few smaller ova (<1.5 cm) that were not counted.

The variation in ovary mass, ovary length and liver mass across TL and maturation stages is shown in Fig 3A to 3C. The categorization of 78 females was: 26 specimens in Stage 1 (1.5–4.1 m TL, nos. 56–81) having poorly developed reproductive organs (S2A and S2B Fig); 44 specimens in Stage 2, 2(7) or 2(?) (4.0–4.9 m TL, nos. 82–125) with enlarged ovaries and for post-natal specimens also enlarged uteri (S3A to S3D Fig and S5A and S5B Fig); seven specimens in Stage 3, 3(7) or 3(?) (4.0–4.7 m TL, nos. 126–132) having ripening/ripe ova (Fig 4A to 4C); no gravid females (Stage 4–6) were available in the material; one female in Stage 7 (4.4 m TL, no. 133) based on flaccid brown ovaries and heavily enlarged uteri internally densely covered with villi (S4A and S4B Fig). Note, that Stage 2(?) and 3(?) refers to four specimens for which previous births could not be established due to lack of data on the uterus (S2 Table). The most distinct parameters between female maturation stages were ovary mass, ova size, and the appearance of the uterus. For females that had given birth previously (Stage 7 and X(7)), the inner epithelium of the heavily enlarged uterus was covered with red/purple villi. These distinct parameters for each maturation stage are quantified in Table 2.

### Aim 2: Body length-at-maturity

**Fig 5:**
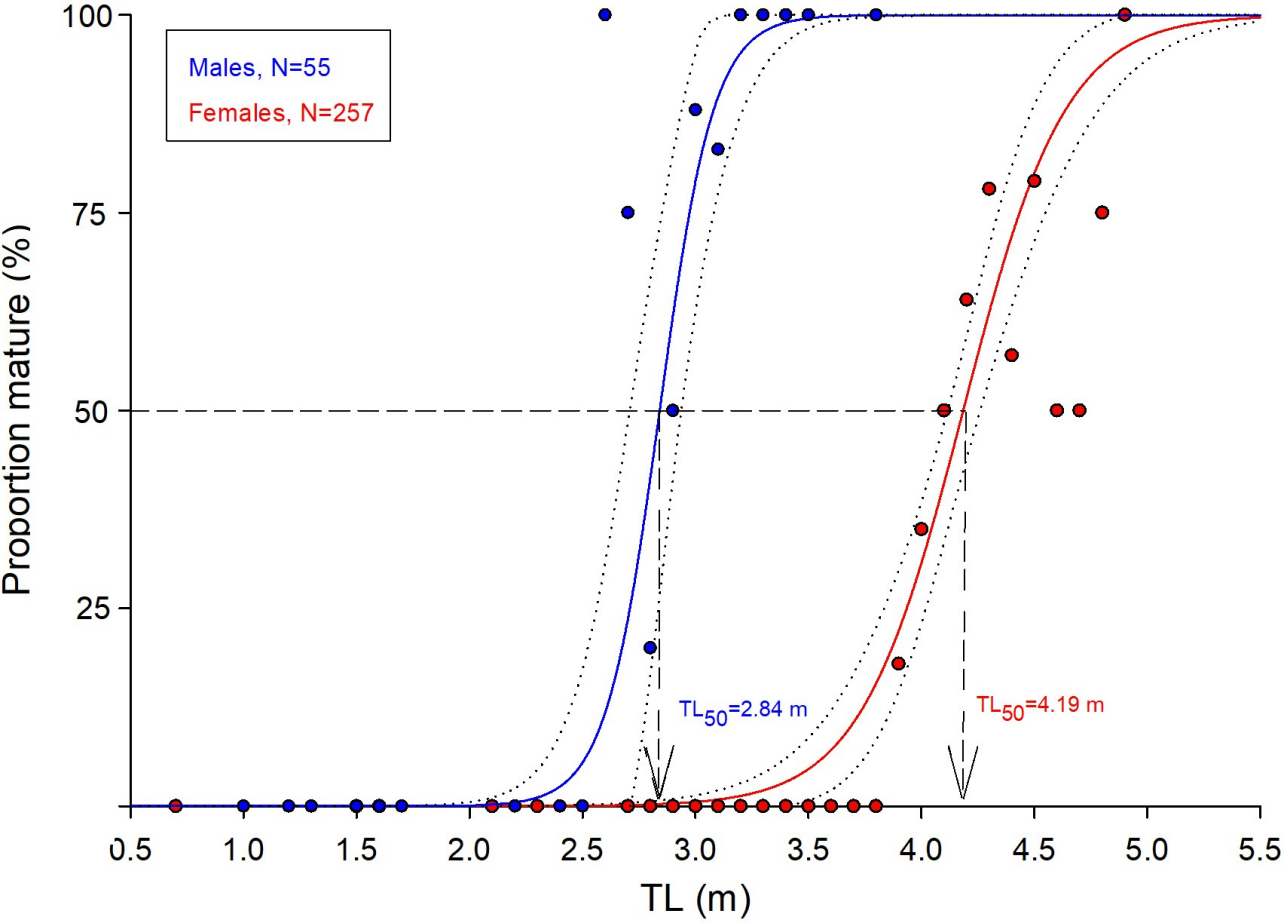
Fitted maturity ogives with 95% confidence limits. Points represent the proportion of mature individuals in each 10 cm bin (males: blue, females: red). Ogives estimate TL_50_ to be 2.84 m for males and 4.19 m for females. Note that for size bins of 4.6 m or larger, sample size is between one and five specimens (see S6 Fig).

To evaluate body length-at-maturity, data encompass all 44 males and 78 females of Aim 1. Of these, 17 males were ‘immature’ and 27 were ‘mature’ (S1 Table), whereas 26 females were ‘immature’ and 52 were ‘mature’. Additional four males (1.5–2.8 m TL), with testes lengths ≤29 cm, were categorized as ‘immature’ (cf. Table 2) and seven males (2.6–3.4 m TL), with testes lengths >30 cm, were categorized as ‘mature’ (cf. Table 2, Fig 2B, S1 Table). Of the 155 females evaluated on-sight by Berland (S3 Table), 102 specimens (3.4–4.7 m TL) were ‘immature’ and 53 were ‘mature’ (3.9–4.9 m TL). Lastly, 23 females (0.7–4.8 m TL) with an ovary mass from 0.1–1.7 kg were categorized ‘immature’ and one (4.5 m TL) with an ovary mass of 2.5 kg was categorized ‘mature’ (Fig 3A, S2 Table). The total dataset of Aim 2 is therefore composed by 21 ‘immature’ males (1.0–3.1 m TL) and 34 ‘mature’ males (2.6–3.8 m TL, S6A Fig), as well as 151 ‘immature’ females (0.7–4.8 m TL) and 106 ‘mature’ females (3.9–4.9 m TL, S6B Fig). Thus, body length-at-first maturity was 2.6 m TL for males and 3.9 m TL for females. The modelled maturity ogives gave an estimated TL_50_ (mean±SE) of 2.84±0.06 m for males and 4.19±0.04 m for females (Fig 5). Ogive coefficients (intercept and STL) were -23.649 and 8.323 for males and -18.424 and 4.402 for females.

### Aim 3: Fecundity, body length-at-birth, and the reproductive cycle

Greenland shark: Ovarian fecundity of three Stage 3 females of 4.0 m, 4.5 m and 4.7 m TL was ~400, 455 and 649 ripe/ripening ova, respectively (Fig 4, nos. 128, 129 and 132 in S2 Table). For the two largest ova counts, the associated ovary masses were 49 and 80 kg. For Shark no. 129, all ~400 ova were about 7.0 cm in diameter. For Shark no. 128 and 132, a subsample of 25 randomly selected ripe/ripening ova were 5.5±0.2 cm (up to 6.0 cm) and 5.1±0.6 cm (up to 7.0 cm) in mean diameter (±SD) (Fig 4). For these two specimens, numerous small ova (<1.5 cm in diameter) were observed but not counted (Fig 4C, J. Nielsen pers. obs.). Ovarian fecundity of one Stage 2 female of 4.3 m TL (no. 92) was 614 ova measuring from 2.1–3.5 cm in diameter. These ova appeared developing and made up ~84% of the ova mass, whereas the remaining ~16% were undeveloped ova measuring from 0.1–2.0 cm in diameter (S7 Fig). Liver mass for all sharks (males and females, Fig 3C) is positively correlated with TL (r = 0.7324, P<0.01, N = 49). When evaluated separately, liver mass is correlated with TL of males (r = 0.8057, P<0.01, N = 16), with the largest liver of 78 kg. Liver mass is also correlated with TL of immature females (r = 0.8424, P<0.01, N = 21) of which the largest liver is 95 kg (Fig 3C). By contrast, mature females exhibit great variation in liver mass (57–274 kg) which is uncorrelated with TL (r = 0.1338, P>0.05, N = 12). Among mature females, the highest liver mass of 274 kg belonged to a Stage 2 female (no. 101), whereas the most depleted liver of 57 kg belonged to a Stage 2(7) female (no. 119, S2 Table). The stage 2 female (no. 92) with 614 developing ova had a liver mass of 236 kg, whereas the Stage 3 female with 649 ripe/ripening ova had a liver mass of 158 kg (no. 128, S2 Table).

**Fig 6:**
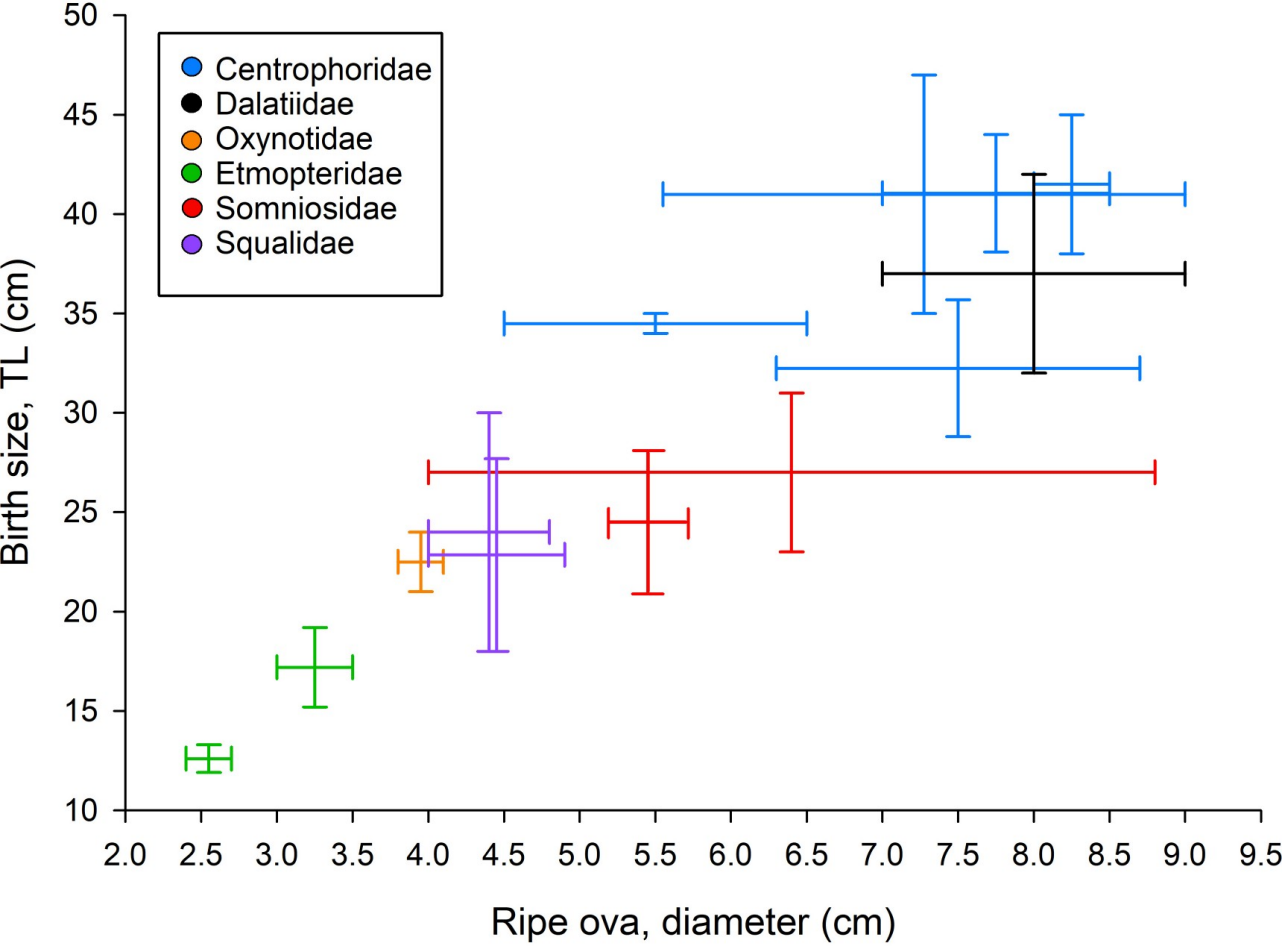
Correlation between size of ripe ova and body length-at-birth. Acquired data on birth size (TL, cm) plotted against size of ripe ova (diameter, cm) for 13 Squaliforms representing six families. Following the order on the X-axis, species are *Etmopertus spinax*, *Centroscyllium fabricii*, *Oxynotus centrina*, *Squalus acanthias*, *Squalus megalops*, *Somniosus rostratus*, *Centrophorus moluccensis*, *Centroscymnus coelolepis*, *Centrophorus granulosus*, *Centrophorus uyato*, *Centrophorus squamosus*, *Dalatias licha*, and *Centrophorus* sp. 1 (see S4 Table).

Other Squaliforms: The reproductive parameters for 23 species are shown in S4 Table. Both ovarian and uterine fecundity vary greatly. Species like *Centrophorus harrisoni*, *Centrophorus moluccensis* and *Squalus megalops* have low fecundities (1–4 pups per pregnancy, [29, 64]), whereas *Scymnodalatias albicauda*, *Echinorhinus brucus* and *Centroscyllium fabricii* have as many as 59, 52 and 35 pups per pregnancy, respectively [27, 41, 53]. There is no correlation between the maximum TL recorded for given species (TL_max_) and the maximum uterine fecundity across the evaluated Squaliforms (Pearson’s correlation test, r = 0.39, P>0.05, N = 20, TL_max_ acquired from Compagno [65] and the referenced literature (cf. S4 Table). Yet, for some species of Centrophoridae (N = 1), Etmopteridae (N = 2), Somniosidae (N = 2) and Squalidae (N = 3), positive correlations between fecundity and maternal size have been reported (S4 Table). The body length-at-birth among the investigated Squaliforms vary from 12–47 cm TL (S4 Table) and is strongly correlated with size of ripe ova which vary from ~2–8 cm in diameter (Fig 6, Pearson’s correlation test, r = 0.93, P<0.01, N = 13, calculated as the correlation between ranges’ mid-point values). For Squaliforms in general, the development of ova may either follow a concurrent cycle, where vitellogenesis occurs parallel to gestation, or it can be non-concurrent where ovaries/uteri enter a resting phase during gestation/vitellogenesis, respectively. Members of Centrophoridae and Squalidae exhibit concurrent cycles, whereas Dalatiidae, Oxynotidae, Etmopteridae, and Somniosidae exhibit non-concurrent development of ova (S4 Table).

The UO_max_-ratios were calculated for 20 species and ranged from 47.1–100%. For Centrophoridae (N = 7) the ratio is 60.0–100%, 77.8% for Dalatiidae (N = 1), 94.7% for Echinorhinidae (N = 1), 47.1–68.1% for Oxynotidae (N = 2), 50.0–76.1% for Etmopteridae (N = 3) and 83.3–100% for both Somniosidae (N = 3) and Squalidae (N = 3) (Table 3).

**Table 3:** Uterine capacity from ovarian observations in other Squaliforms.

****Species****	****Family****	****N**_**females**_**	****UO**_**max**_**	****%****	****References****
*Centrophorus squamosus* Δ	Centrophoridae	467	10/15	66.7	[35, 36]
*Centrophorus granulosus*	Centrophoridae	156	6/10	60.0	[36]
Centrophorus harrisoni*	Centrophoridae	86	2/2	100	[29]
Centrophorus moluccensis*	Centrophoridae	68	2/2	100	[29]
*Centrophorus* sp. 1	Centrophoridae	100	1/1	100	[29]
*Centrophorus* sp. 2*	Centrophoridae	51	2/3	66.7	[32]
*Deania profundorum*	Centrophoridae	351	11/14	78.6	[37]
Dalatias licha*	Dalatiidae	67	14/18	77.8	[39]
*Echinorhinus brucus*	Echinorhinidae	256	36/38	94.7	[42]
*Oxynotus bruniensis* *	Oxynotidae	45	8/17	47.1	[46]
*Oxynotus centrina* *Δ	Oxynotidae	20	15/22	68.1	[43, 44]
*Centroscyllium fabricii*	Etmopteridae	1476	35/47	74.4	[27]
*Etmopterus granulosus*	Etmopteridae	492	15/30	50.0	[47]
*Etmopterus spinax*	Etmopteridae	485	16/21	76.1	[49]
*Centroscymnus coelolepis*	Somniosidae	871	25/30	83.3	[52]
*Centroscymnus owstoni*	Somniosidae	316	28/28	100	[51]
Somniosus rostratus*	Somniosidae	29	21/23	91.3	Guallart unpublished data
*Squalus acanthias*	Squalidae	491	15/18	83.3	[57]
*Squalus megalops*	Squalidae	4180	4/4	100	[64]
*Squalus suckleyi* ○	Squalidae	360	16/13	100	[55]

* Sample sizes low compared to other studies (N>100).

Δ Ratio calculated on numbers from different studies but from same region.

○ Uterine fecundity is higher than ovarian fecundity.

Maximum number of fetuses over maximum number of ripe ova, i.e. UO_max_-ratio (expressed as %). This ratio reflects the species’ maximum capacity uterine fecundity (U) based on ovarian observations (O).

The ovarian fecundity of Greenland shark ranges from 400–649 ova (N = 4). For several Squaliforms including other members of Somniosidae maternal size and fecundity are positively correlated but the correlation is not supported by all studies within the same species (S4 Table). It is plausible, however, that the fecundity of Greenland sharks is correlated with maternal size and that fecundity of the largest females (TL >5 m) can be higher than documented in this study. Although UO_max_-ratios for Somniosidae such as *Centroscymnus coelolepis*, *Centroscymnus owstoni* and *Somniosus rostratus* are >80%, we conservatively estimate the uterine fecundity of Greenland shark to be at least ~50% of the observed ovarian fecundity. This estimate is based on the lowest ratio found among the investigated Squaliforms (Table 3). The maximum uterine fecundity for Greenland sharks (or the capacity of the uterus) is therefore estimated to between 200 and 324 pups per pregnancy depending on maternal size–and possibly higher for females near maximum size. Future observations of gravid females is, however, expected also to reveal a high variation in uterine fecundity similar to e.g. the black dogfish (see [27]).

The largest ripe ova observed in this study were 7.0 cm, whereas Nikolsky [11] reported ova up to 8.0 cm in diameter. For Greenland sharks, the size range of ova ready for ovulation is unknown but when comparing size of ripe ova and body length-at-birth for other Squaliforms (Fig 6), its seems that Greenland shark ripe ova of ~7–8 cm, would likely develop into pups of 35–45 cm TL. Such body length-at-birth corresponds well with the near-term Greenland shark fetuses of 37 cm [13] as well as with the smallest free-swimming specimens of 41.8–46.7 cm TL (N = 4, [66, 67]).

## Discussion

We provide the first statistical analyses of body length-at-maturity, quantitative descriptions of reproductive parameters as well as estimate fecundity, body length-at-birth and elucidate the reproductive cycle of the Greenland shark.

### Body length-at-maturity and maturation stages

For Greenland sharks, females attain larger size than males. The largest verified male measured 3.8 m TL (this study) whereas the largest known female measured 5.5 m TL [1, 68]. We find, that body length-at-first maturity for males is 2.7 m TL and TL_50_ 2.86 m, which is less than that suggested by Yano et al. [8]. For females, body length-at-first maturity is found to be 3.9 m TL and TL_50_ 4.19 m, revealing that Greenland shark females not only attain greater maximum size, but also mature at greater lengths than males. The lifespan of Greenland shark females was estimated by Nielsen et al. [2] and, combined with body length-at-maturity presented here, suggests that females reach sexual maturity no earlier than 134 years of age. We emphasize, however, that due to the new Marine20 calibration curve [69], age estimates are currently being reevaluated (Olsen et al. in prep). For males, the lack of age estimates precludes any inferences about age-at-maturity. Morphological parameters allowing for identification of reproductive stages of both males and females are presented in the guidelines in Table 2. We recommend that these are used for future categorization of maturation stage and that they are continuously improved with more data. From our findings, it is evident that although females display no discernable external signs, specimens of <3.9 m TL can fairly be considered as immature (Stage 1). For potentially mature females (>3.9 m TL), their specific maturation stages can only be assessed from inspection of ovaries and uteri. Ideally, such data will be acquired using non-lethal ultrasonic scanners for reproductive assessment prior to release [70].

### Fecundity, body length-at-birth, and the reproductive cycle

Under the assumption of correct species identification in the original reports, the review paper by MacNeil et al. [7] presents a birth size range of 40–100 cm TL and a fecundity of at least 10 pups for Greenland sharks. These ranges are also referenced by e.g. ICES [71] and Edwards et al. [5]. However, when critically reviewing the original reports behind these numbers, some information must be considered doubtful. For example, although species identification is validated for the gravid female reported by Koefoed [13] with 10 near-term pups of 37 cm TL, it was caught on a longline of unknown soaking time (during targeted Greenland shark fishery, [72]). Capture-induced abortion could thus have caused underestimation of the actual fecundity—a potential bias often mentioned in the scientific literature on reproductive biology of Squaliforms (see [23, 39, 42, 57, 60, 61, 64]). Another possibility is that the gravid female gave birth to the majority of the batch in the recent past and the 10 pups thus were the last ones remaining in the uterus. Nevertheless, a uterine fecundity of only 10 pups seems too low based on the available ovarian data from Greenland sharks as well as the phylogenetic comparison of this study. Another report suggesting even lower uterine fecundity and a very large body length-at-birth, is Bjerkan [14] with a single ~98 cm Greenland shark fetus from a female caught in a fjord in western Norway. Bjerkan did not, however, confirm the fisherman’s species identification and because the similar-sized *Cetorhinus maximus* (Lamniformes) throughout history has been misidentified as a Greenland shark (in local media in both Iceland, Norway and Greenland, see [68]), this report could be such a case. As for other Lamniforms with a oophagous reproductive strategy, *Cetorhinus maximus* supposedly produces few but large pups as other members of the order [73, 74]. The last report suggesting a very large body length-at-birth of Greenland sharks is by Kondyurin & Myagkov [75] with two free-swimming specimens of ~1 m. Due to “the presence of yolk-like material in the guts” (translated from Russian) these two were estimated to be 10–15 days old (i.e. neonates). Kondyurin & Myagkov [75] further question the validity of Nikolsky’s statement of ~500 large ova, see Introduction). However, our data support Nikolsky and therefore, we counterargue that the two ~1 m specimens reported by Kondyurin & Myagkov [75] were not neonates. Moreover, at the Natural History Museum of Denmark one Greenland shark of ~60 cm TL and another of 81 cm TL [2] was visually inspected and carried no signs of being neonates (e.g. umbilical scar or yolk sack remnants, J. Nielsen pers obs.). Lastly, from what is known about Chondrichthyan reproductive modes it is difficult to imagine a strategy that, within the same species, would yields pups varying as much as 40 cm to 100 cm TL in body length-at-birth.

We argue that Greenland shark can produce much more pups per pregnancy than previously believed (uterine fecundity >200) and that the body length-at-birth is relatively small (35–45 cm TL) compared to female body size. We hypothesize that future observations of gravid Greenland sharks will reveal such high uterine fecundity, which also will be widely scattered around an overall positive relationship between maternal length and fecundity, similar to the well-documented trend for *Centroscyllium fabricii* (see Fig 10 in Yano [27]).

To elucidate additional aspects of the reproductive cycle of the Greenland shark, original and phylogenetic data are evaluated. It is common for Squaliforms to invest large amounts of energy into reproduction. For *Centrophorus squamosus*, *Centroscymnus coelolepis* and *Centroscyllium fabricii*, ripe ova constitute as much as 7.5%, 15% and 22% of the body mass, respectively [27, 31]. In comparison, two Stage 3 females in our study weighed 1,100 kg and 1,367 kg (no. 132 and 128, S2 Table), and hence their ova constituted ~4.5% and ~5.9% of the body mass. However, for these two females, all ova were not necessarily fully mature (i.e. some might be ripening instead of ripe) and the proportion of ova mass to body mass (as presented above), might not reflect their full reproductive investment. Another indicator of the large energy investment of Greenland sharks into reproduction, is the observed variation in liver mass among mature females (range 57–272 kg, Fig 3C). The main components of yolk derive from hepatic proteins [76] and hence, the high variation in liver mass can be associated with the large production of ripe ova. The largest livers will thus typically be observed in Stage 2 females in early vitellogenesis (e.g. no. 92 with 614 developing ova from 2.1–3.5 cm and a liver of 236 kg), whereas Stage 3 females (ripening and ripe) gradually have a more depleted liver (e.g. no. 132 with 649 ova of 5.0–6.0 cm and a liver of 158 kg). Post-natal females in Stage 7 are expected to have the most depleted livers, such as no. 199 with a liver of 57 kg (S2 Table).

The reproductive development of Greenland sharks is supposedly cyclic (see reproductive cycle e.g. for *Centroscyllium fabricii* in Yano [27] or *Centroscymnus coelolepis* in Clarke et al. [31]), and the liver mass of mature Greenland shark females is thus expected to increase as post-natal females re-enter the developing Stage 2. The duration of building up liver mass prior to vitellogenesis, the duration of the actual vitellogenesis as well as gestation time is unknown. However, for some Squaliforms gestation times are 1 to 2 years [39, 43, 57, 61] and as much as 3.5 years for the *Chlamydoselachus anguineus* (Hexanchiformes, [77]). Augustine et al. [78] estimated the gestation time for Greenland sharks to be 8–18 years but as several inputs to the applied model are questionable (see discussion on species’ maximum size in Nielsen [68] and fecundity in this study), we consider that gestation time for the Greenland shark is unknown.

As for other species of Somniosidae (and the majority of Squaliform families, S4 Table), it is likely that Greenland sharks exhibit a non-concurrent development of ova. This means, that ovaries and uteri rest alternately, and that ripe ova are either fertilized (and transferred to the uteri) or reabsorbed in the ovaries. Non-fertilized degenerating ova are referred to as atretic. No atretic ova were found in post-natal females (J. Nielsen pers obs), and ovarian atresia is known only from few unquantified observations (B. Berland pers. obs.). Therefore, we consider it plausible, that the number of atretic ova in Greenland sharks is relatively low (and not counting into the hundreds). Atretic ova are commonly reported for Squaliforms including all three Somniosidae species in the phylogenetic comparison (Aim 3). Their presence in ovaries of post-natal Greenland sharks is therefore expected. The relatively low number of atretic ova among non-concurrent Squaliforms is also reflected in the little difference between mean ovarian and mean uterine fecundity (see S4 Table).

A recent review speculate if the Greenland shark uterus can support the respiratory demands of high fecundities [79]. However, we conservatively estimate the capacity of the Greenland shark uterus to be at least 200–324 pups per pregnancy based on multiple observations of high ovarian fecundity (400–649 ova). Similar high ovarian fecundities are known also for the large-bodied and closely related *Somniosus pacificus* [80, 81]. Body length-at-birth for *Somniosus pacificus* is supposedly also relatively small (~40 cm TL [8]) and all combined, we propose that the estimates on capacity of the Greenland shark uterus may also apply for the two other large members of the *Somniosus* genus being *Somniosus antarticus* and *Somniosus pacificus* (see [8, 82]).

Overall, the findings presented here on Greenland shark reproductive biology are relevant for future research efforts trying to identify mating locations, pupping and nursery areas as well as better interpret the complex migration and spatial behavior already documented for the species (see [1, 83, 84]). Therefore, we believe this study will contribute to improved management and conservation of a long-lived top predator which manifest great resilience despite a history of heavy fisheries exploitation and unwanted bycatch [5]. Data of gravid females are needed though to validate and more accurately describe the full reproductive capacity of Greenland shark, which likely could be the highest among Chondrichthyans.

## Supporting information

S1 Fig Male reproductive organs. (a+b) testes and clasper from immature male. (c+d) testes and clasper from mature male. (e+f) extruded clasper spur.
(TIF)

S2 Fig Reproductive organs of Stage 1 female. (a+b) poorly developed ovaries and uteri of an immature female.
(JPG)

S3 Fig Reproductive organs of Stage 2 female. (a) developing ovaries and poorly developed uteri of mature female in early vitellogenesis. (b) as vitellogenesis proceeds, size of ova increase as does ovary mass. (c+d) uteri increasing in size yet remain non-covered with villi internally. Oviduct is highlighted for overview of the anatomy.
(JPG)

S4 Fig Reproductive organs of Stage 2 (7) female. (a-c) developing ovaries and enlarged uteri of a post-natal female. (d+e) during vitellogenesis size of ovaries increase and uteri remain internally are covered with villi from previous pregnancy. Liver, stomach and oviduct is highlighted for overview of the anatomy.
(JPG)

S5 Fig Reproductive organs of a Stage 7 female. (a) exhausted ovaries from post-natal female that has not re-entered developing stage. (b) uteri enlarged and covered internally with dense layer of villi.
(JPG)

S6 Fig Composition of immature and mature specimens. Size composition of (a) males and (b) females for sharks of Aim 2.
(JPG)

S7 Fig Composition of developing ova during vitellogenesis. Weight and number (above bars) of 1,327 ova from 0.1–3.5 cm (diameter) from the ovary of a developing Stage 2 female (no 92). The majority of the ova mass was comprised by 614 developing ova from 2.1–3.5 cm. The left ovary contained the highest number of ova.
(JPG)

S1 Table Reproductive parameters of male sharks. Individual data available for all males analyzed. Each specimen has a unique shark identification number. For nos. 1–44 at least two parameters were available assigning maturity stage (M. stage), whereas only testes length was available for nos. 45–55. These 11 sharks were categorized (Cat.) as ‘immature’ or ‘mature’ for Aim 2, based on findings of Aim 1. Source refers to either J. Nielsen (JN), K. Yano (KY) or Bjørn Berland (BB) as the collector of data.
(DOCX)

S2 Table Reproductive parameters of female sharks. Individual data available for all female sharks analyzed. Each specimen has a unique shark identification number which are continuous from previous S1 Table. ‘Ova stage’ is either ‘Nd’ = not developed, ‘Gran.’ = granulated, ‘Dif.’ = Differentiated or ‘Ripe’. ‘Ova max. dia’ refers to maximum diameter of ova. For nos. 134–157, only relatively low ovary mass were available (<2.5 kg) from which these were categorized as ‘immature’ or ‘mature’ in Aim 2 based on findings of Aim 1. ‘M. stage’ refers to maturity stage. Source refers to either J. Nielsen (JN), K. Yano (KY) or Bjørn Berland (BB) as the collector of data.
(DOCX)

S3 Table Berland’s additional sharks. Female sharks categorized on sight as either ‘Immature’, ‘Maturing’ or ‘Mature’ by Berland. Shark identification numbers are continuous from previous S1 and S2 Tables.
(DOCX)

S4 Table Reproductive parameters of Squaliforms. Reproductive strategy (Repr. strat.) can either be Lec = Lecitotrophic, Ma = Matrotrophic or UA = Unassessed. Mean values are in []. Ovarian cycle is either concurrent (conc.) or non-concurrent (N-conc.). Abbreviations for regions are: Atl. = Atlantic Ocean, Med. = Mediterranean, Pac. = Pacific, Indi. = Indian Ocean, Arab. = Arabian Sea, Bl. Sea = Black Sea.
(DOCX)
