# Supplementary figures and images for: Assessing the reproductive biology of the Greenland shark (Somniosus microcephalus)
Source: PLoS One. 2020 Oct 7;15(10):e0238986. doi: 10.1371/journal.pone.0238986 (PMC7540863; doi:10.1371/journal.pone.0238986)

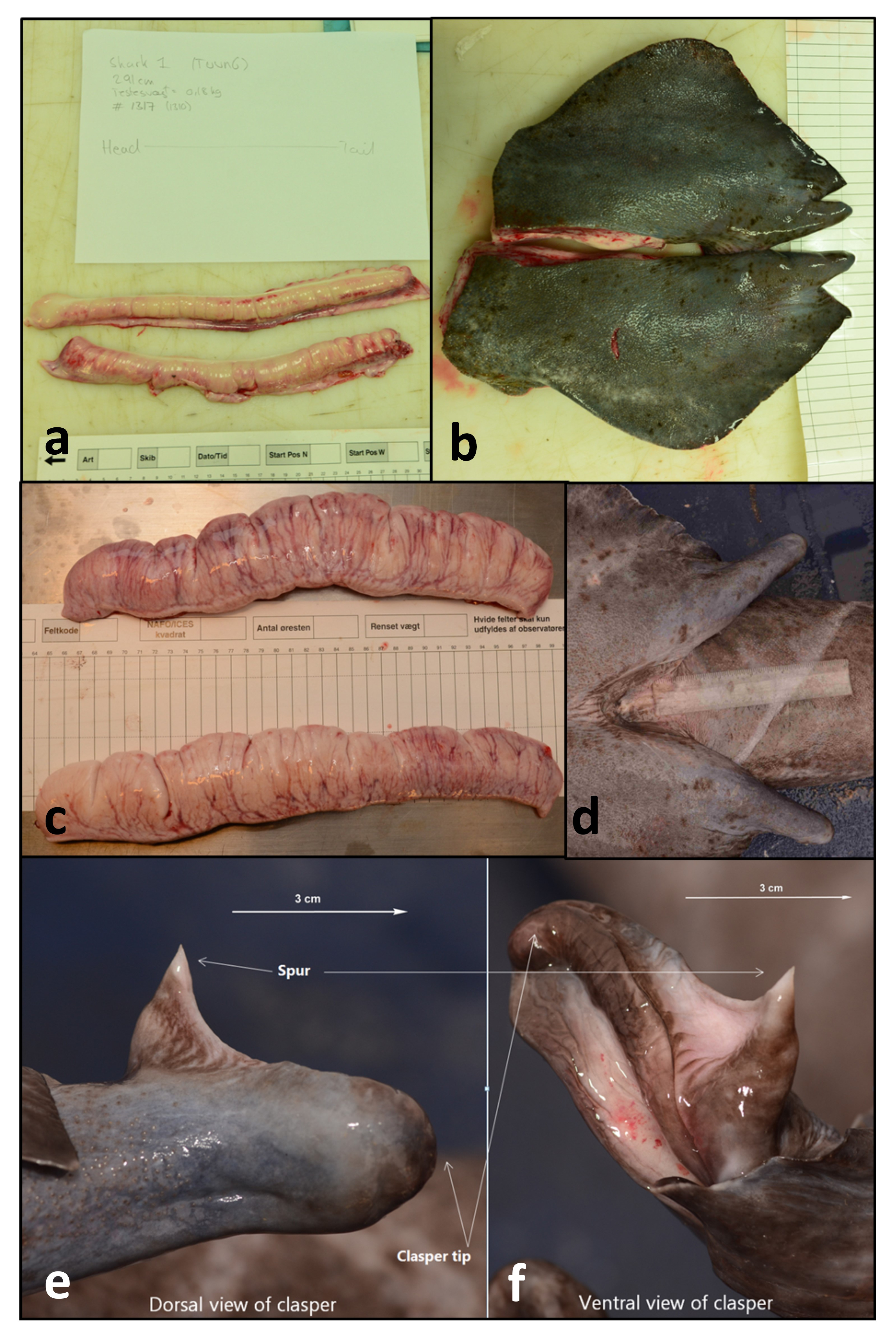

Supplement: S1 Fig — (a+b) testes and clasper from immature male. (c+d) testes and clasper from mature male. (e+f) extruded clasper spur. (TIF) [file pone.0238986.s001.tif]

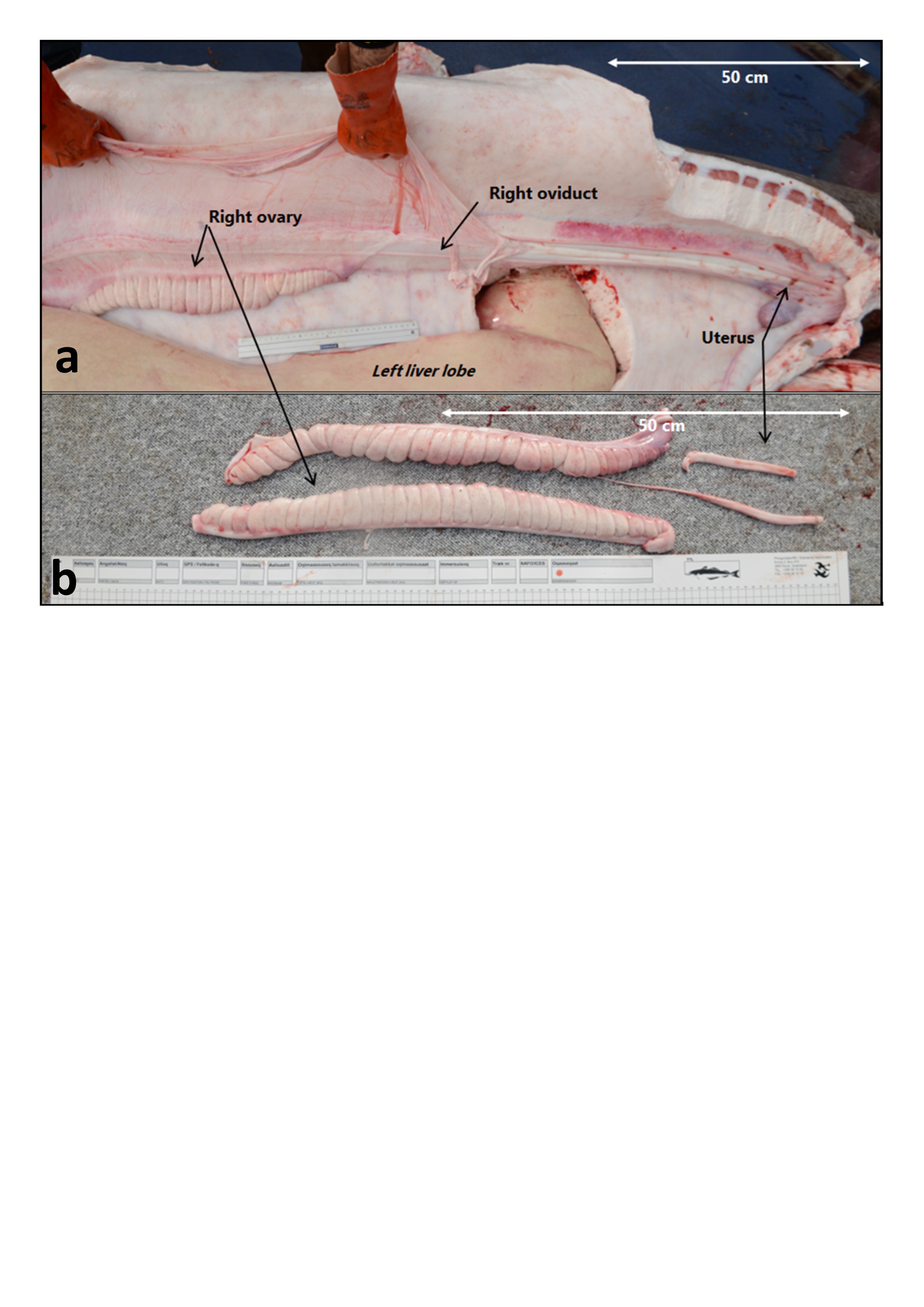

Supplement: S2 Fig — (a+b) poorly developed ovaries and uteri of an immature female. (JPG) [file pone.0238986.s002.jpg]

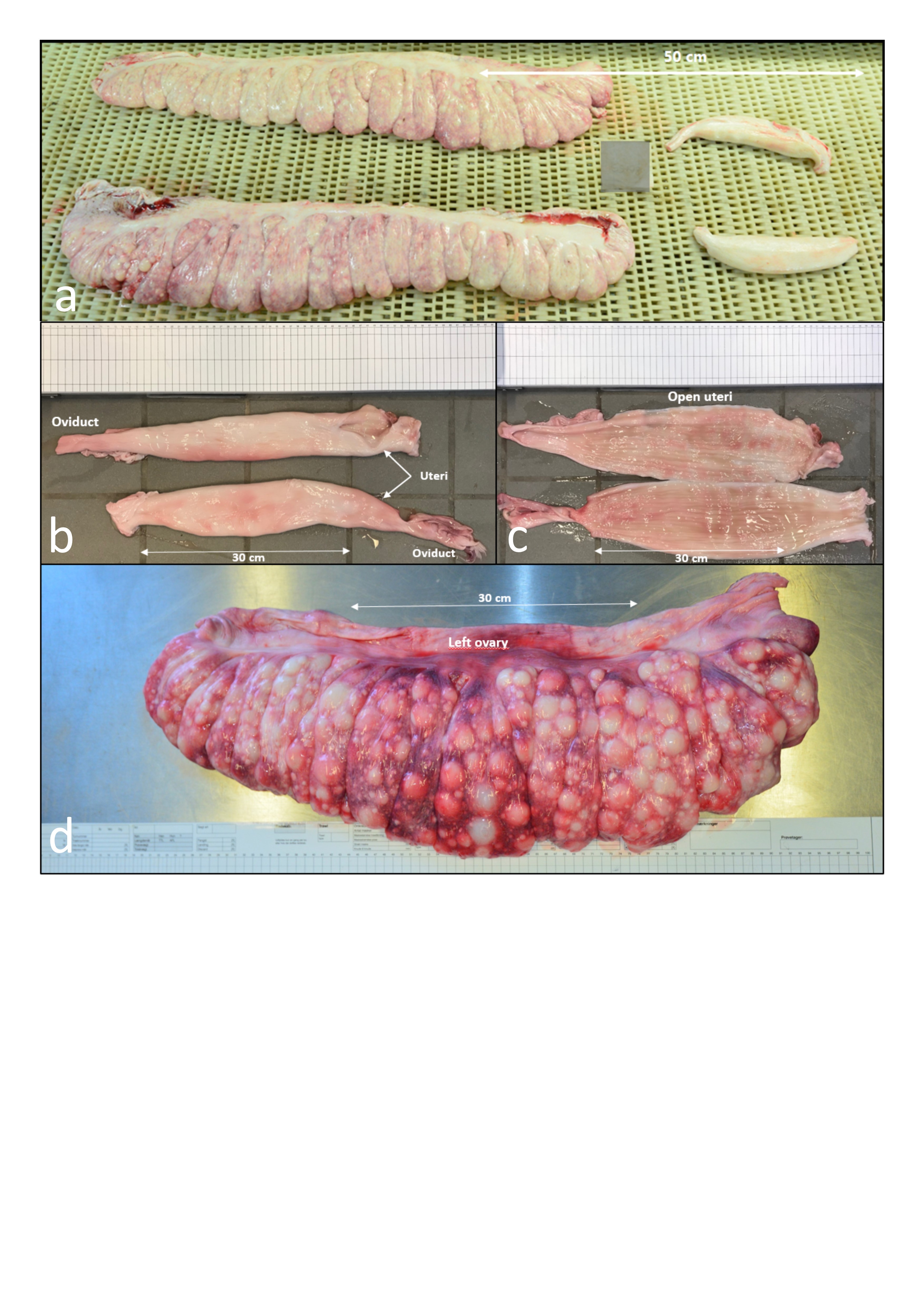

Supplement: S3 Fig — (a) developing ovaries and poorly developed uteri of mature female in early vitellogenesis. (b) as vitellogenesis proceeds, size of ova increase as does ovary mass. (c+d) uteri increasing in size yet remain non-covered with villi internally. Oviduct is highlighted for overview of the anatomy. (JPG) [file pone.0238986.s003.jpg]

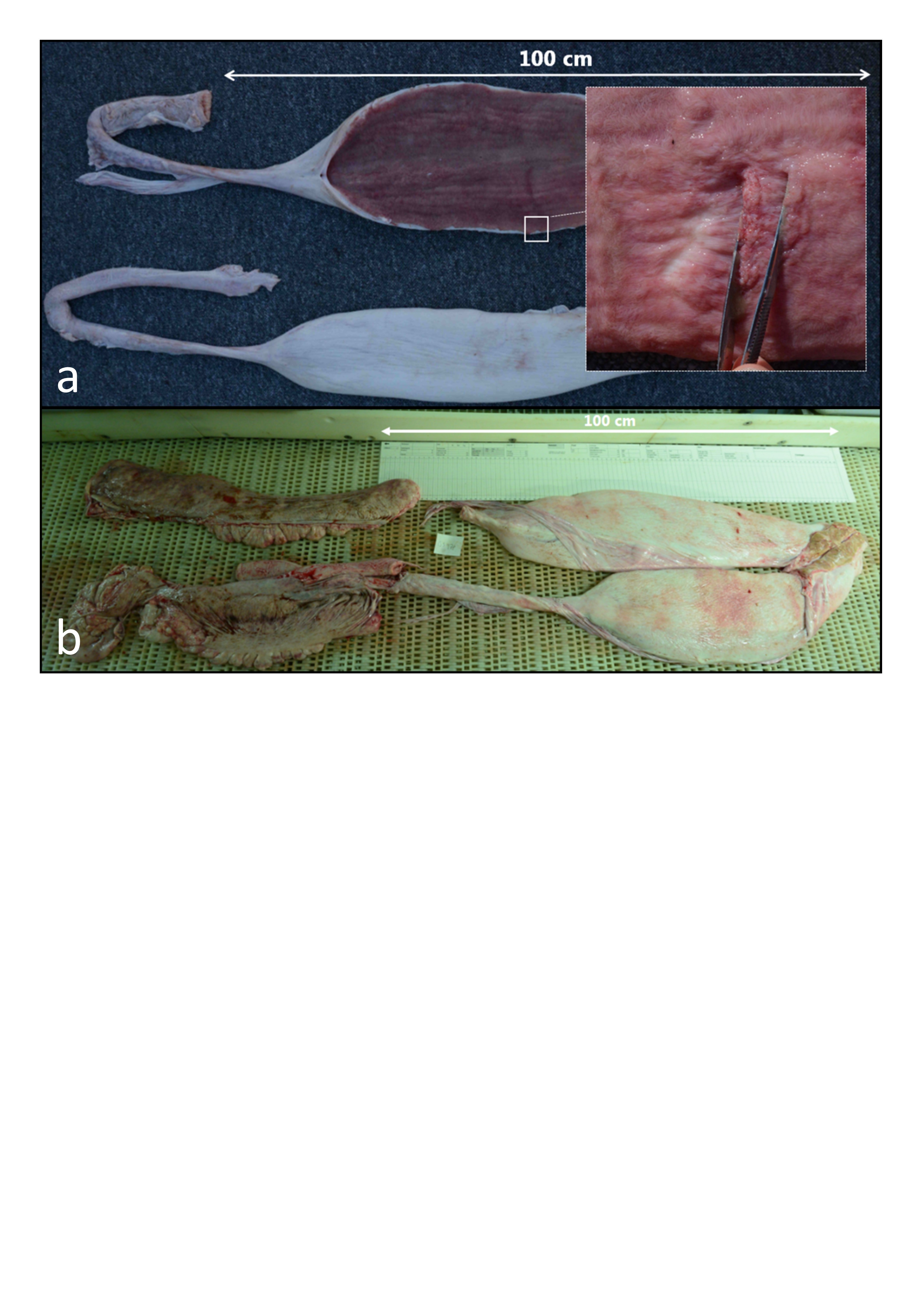

Supplement: S4 Fig — (a-c) developing ovaries and enlarged uteri of a post-natal female. (d+e) during vitellogenesis size of ovaries increase and uteri remain internally are covered with villi from previous pregnancy. Liver, stomach and oviduct is highlighted for overview of the anatomy. (JPG) [file pone.0238986.s004.jpg]

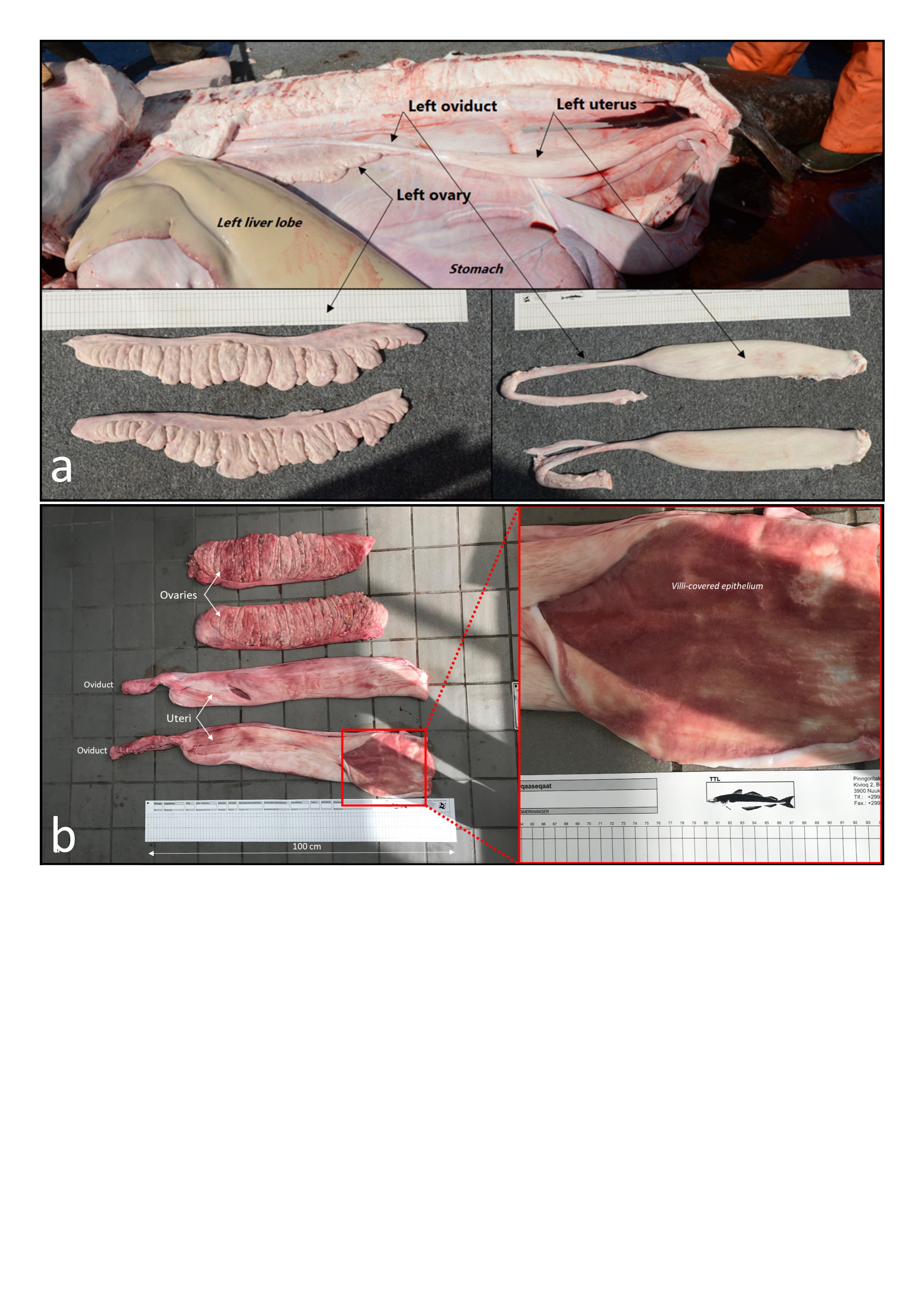

Supplement: S5 Fig — (a) exhausted ovaries from post-natal female that has not re-entered developing stage. (b) uteri enlarged and covered internally with dense layer of villi. (JPG) [file pone.0238986.s005.jpg]

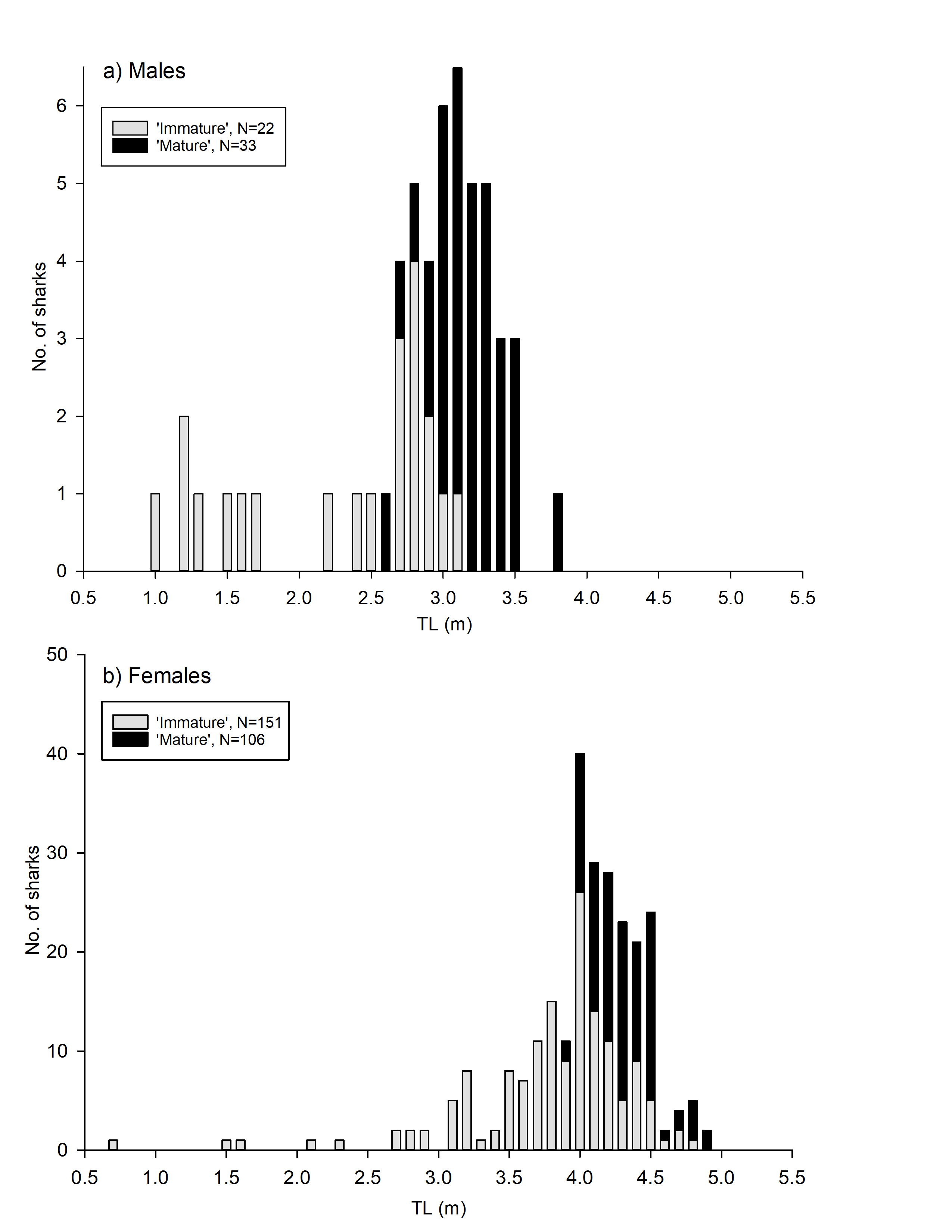

Supplement: S6 Fig — Size composition of (a) males and (b) females for sharks of Aim 2. (JPG) [file pone.0238986.s006.JPG]

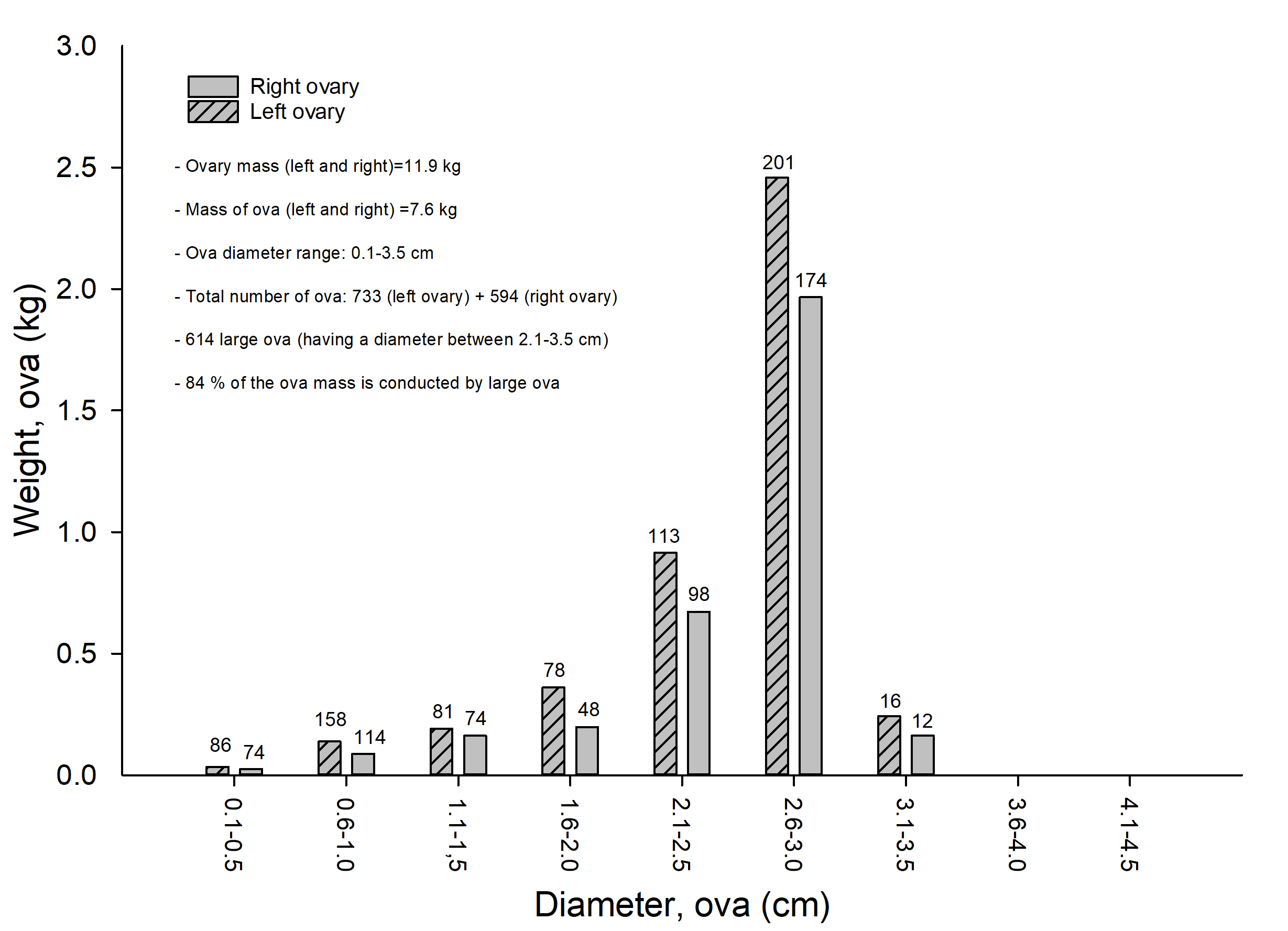

Supplement: S7 Fig — Weight and number (above bars) of 1,327 ova from 0.1–3.5 cm (diameter) from the ovary of a developing Stage 2 female (no 92). The majority of the ova mass was comprised by 614 developing ova from 2.1–3.5 cm. The left ovary contained the highest number of ova. (JPG) [file pone.0238986.s007.JPG]
