# Supplementary material for: Assessing the reproductive biology of the Greenland shark (Somniosus microcephalus)
Source: PLoS One. 2020 Oct 7;15(10):e0238986. doi: 10.1371/journal.pone.0238986 (PMC7540863; doi:10.1371/journal.pone.0238986)
Supplement: S1 Table — Individual data available for all males analyzed. Each specimen has a unique shark identification number. For nos. 1–44 at least two parameters were available assigning maturity stage (M. stage), whereas only testes length was available for nos. 45–55. These 11 sharks were categorized (Cat.) as ‘immature’ or ‘mature’ for Aim 2, based on findings of Aim 1. Source refers to either J. Nielsen (JN), K. Yano (KY) or Bjørn Berland (BB) as the collector of data. (DOCX) [file pone.0238986.s008.docx]

**S1 Table (1 of 2)**.

| **No.** | **TL** | **Liver** | **Testes** | | | | **Claspers** | | | | **Sperm** | **M. stage** | **Source** |  |
| --- | --- | --- | --- | --- | --- | --- | --- | --- | --- | --- | --- | --- | --- | --- |
|  | (m) | (kg) | Mass (kg) | Length (cm) | Color | Form | Spur | Rigidity | Tip beyond fin | Length (cm) |  |  |  |  |
| 1 | 1.0 | 0.9 | <0.01 |  | White |  |  | Soft | No |  |  | 1 | JN |  |
| 2 | 1.6 | 3.2 | <0.01 |  | white |  |  | Soft | No |  |  | 1 | JN |  |
| 3 † | 2.7 | 24.8 | 0.1 |  | White/pink | Thin | No | Soft | No | 12 |  | 1 | JN |  |
| 4 | 2.8 | 20.0 | 0.2 |  |  |  |  |  |  |  |  | 1 | JN |  |
| 5 † | 2.9 | 27.2 | 0.2 | 28 | White/pink | Thin | No | Soft | No | 11 |  | 1 | JN |  |
| 6 | 1.2 | 0.8 | <0.01 |  | White | Thin |  | Soft | No |  |  | 1 | JN |  |
| 7 | 3.1 | 36.0 | 0.1 | 29 | Reddish | Thin |  |  |  |  |  | 1 | JN |  |
| 8 | 1.2 |  | 0.01 |  |  |  |  | Soft |  | 6 |  | 1 | KY |  |
| 9 | 1.3 |  | 0.01 |  |  |  |  | Soft |  | 5 |  | 1 | KY |  |
| 10 | 2.2 |  | 0.1 |  |  |  |  | Soft |  | 11 |  | 1 | KY |  |
| 11 | 2.4 |  | 0.2 |  |  |  |  | Soft |  | 13 |  | 1 | KY |  |
| 12 | 2.5 |  | 0.2 |  |  |  |  | Soft |  | 14 |  | 1 | KY |  |
| 13 | 2.7 |  | 0.2 |  |  |  |  | Soft |  | 17 |  | 1 | KY |  |
| 14 | 2.7 |  | 0.2 |  |  |  |  | Soft |  | 16 |  | 1 | KY |  |
| 15 | 2.8 |  | 0.2 |  |  |  |  | Soft |  | 18 |  | 1 | KY |  |
| 16 | 2.8 |  | 0.2 |  |  |  |  | Soft |  | 17 |  | 1 | KY |  |
| 17 | 2.9 |  | 0.2 |  |  |  |  | Soft |  | 16 |  | 1 | KY |  |
| 18 † | 2.7 | 32.4 | 0.2 | 31 | White/pink | Thin |  | Soft | Yes | 17 |  | 2 | JN |  |
| 19 | 2.9 |  |  |  | white | Indented |  | Soft | Yes |  |  | 2 | JN |  |
| 20 | 3.0 |  | 0.4 |  |  |  |  | Soft |  | 17 |  | 2 | KY |  |
| 21 | 2.9 |  | 0.7 | 35 | White/reddish | Indented | Yes | Calcified | Yes | 22 |  | 3 | JN |  |
| 22 †† | 3.0 | 10.5 | 0.7 | 39 | White/pink | Indented | Yes | Calcified | Yes | 20 | Yes | 3 | JN |  |
| 23 | 3.2 |  |  | 40 | White | Indented |  | Calcified | Yes |  |  | 3 | JN |  |
| 24 ∆ | 3.0 |  |  |  |  |  | Yes | Calcified | Yes | 24 |  | 3 | JN |  |
| 25 ∆ | 3.1 |  |  |  |  |  | Yes | Calcified | Yes | 24 |  | 3 | JN |  |
| 26 | 3.1 | 46.3 | 0.70 |  |  |  | No | Calcified |  | 19 | No | 3 | JN |  |
| 27 ● | 3.1 |  |  |  |  |  | Yes | Calcified | Yes | 21 | Yes | 3 | JN |  |
| 28 | 3.2 | 60.0 | 0.6 |  |  |  |  |  |  |  |  | 3 | JN |  |
| 29 | 3.2 | 49.0 | 0.7 | 34 | White/pink | Indented | No | Calcified | Yes | 24 | No | 3 | JN |  |
| 30 | 3.3 | 51.1 | 0.5 | 33 | White | Indented |  |  |  | 23 |  | 3 | JN |  |
| 31 | 3.3 | 39.4 | 0.9 |  |  |  |  |  |  |  |  | 3 | JN |  |
| 32 †† | 3.3 | 78.0 | 1.3 | 53 | Reddish | Indented | Yes | Calcified | Yes |  | Yes | 3 | JN |  |
| 33 ● | 3.4 |  |  |  |  |  |  | Calcified | Yes | 25 |  | 3 | JN |  |
| † Histological examinations of testes support immature shark (L. M. McClusky unpublished data), †† Histological examination suggest mature shark (L. M. McClusky unpublished data), ∆ Shark released and sexual maturation evaluated solely on presence of well-developed and calcified claspers reaching beyond pelvic fin tip, ● Shark caught as carcass causing some measures not to be available | | | | | | | | | | | | | | |

**S1 Table (2 of 2)**.

| **No.** | **TL** | **Liver** | **Testes** | | | | **Claspers** | | | | **Sperm** | **M. stage** | **Source** |  |
| --- | --- | --- | --- | --- | --- | --- | --- | --- | --- | --- | --- | --- | --- | --- |
|  | (m) | (kg) | Mass (kg) | Length (cm) | Color | Form | Spur | Rigidity | Tip beyond fin | Length (cm) |  |  |  |  |
| 34 ∆ | 3.5 |  |  |  |  |  |  | Calcified | Yes |  |  | 3 | JN |  |
| 35 | 3.5 | 65.1 | 0.5 | 30 | White/pink | Indented |  | Deform‡ | Yes | 20 |  | 3 | JN |  |
| 36 | 3.0 |  |  |  |  |  |  | Calcified | Yes | 29 |  | 3 | JN |  |
| 37 | 3.2 |  |  |  |  |  |  | Calcified | Yes |  |  | 3 | JN |  |
| 38 ∆ | 3.3 |  |  |  |  |  |  | Calcified | Yes |  |  | 3 | JN |  |
| 39 ∆ | 3.4 |  |  |  |  |  |  | Calcified | Yes |  |  | 3 | JN |  |
| 40 ∆ | 3.5 |  |  |  |  |  |  | Calcified | Yes |  |  | 3 | JN |  |
| 41 ∆ | 3.8 |  |  |  |  |  |  | Calcified | Yes |  |  | 3 | JN |  |
| 42 | 2.8 |  |  | 32 |  |  |  |  | Yes | 22 |  | 3 | BB |  |
| 43 | 3.1 |  |  | 37 |  |  |  |  | Yes | 22 |  | 3 | BB |  |
| 44 | 3.2 |  | 1.8 |  |  |  |  | Calcified |  | 29 |  | 3 | KY |  |
| 45 | 1.5 |  |  | 16 |  |  |  |  |  |  |  | Cat. ‘immature’ | BB |  |
| 46 | 1.7 |  |  | 13 |  |  |  |  |  |  |  | Cat. ‘immature’ | BB |  |
| 47 | 2.8 |  |  | 27 |  |  |  |  |  |  |  | Cat. ‘immature’ | BB |  |
| 48 | 3.0 |  |  | 35 |  |  |  |  |  |  |  | Cat. ‘mature’ | BB |  |
| 49 | 3.0 |  |  | 35 |  |  |  |  |  |  |  | Cat. ‘mature’ | BB |  |
| 50 | 3.2 |  |  | 31 |  |  |  |  |  |  |  | Cat. ‘mature’ | BB |  |
| 51 | 3.3 |  |  | 36 |  |  |  |  |  |  |  | Cat. ‘mature’ | BB |  |
| 52 | 3.4 |  |  | 40 |  |  |  |  |  |  |  | Cat. ‘mature’ | BB |  |
| 53 | 3.1 |  |  | 47 |  |  |  |  |  |  |  | Cat. ‘mature’ | BB |  |
| 54 | 2.6 |  |  | 31 |  |  |  |  | No |  |  | Cat. ‘mature’ | BB |  |
| 55 | 3.0 |  |  | 23 |  |  |  |  | Yes |  |  | Cat. ‘immature’ | BB |  |
| ∆ Shark released and sexual maturation evaluated solely on presence of well-developed and calcified claspers reaching beyond pelvic fin tip  ‡ Deform claspers refer to one clasper being shorter and the other longer than pelvic fin. The longest clasper was calcified. | | | | | | | | | | | | | | |
