# Supplementary material for: Assessing the reproductive biology of the Greenland shark (Somniosus microcephalus)
Source: PLoS One. 2020 Oct 7;15(10):e0238986. doi: 10.1371/journal.pone.0238986 (PMC7540863; doi:10.1371/journal.pone.0238986)
Supplement: S2 Table — Individual data available for all female sharks analyzed. Each specimen has a unique shark identification number which are continuous from previous S1 Table. ‘Ova stage’ is either ‘Nd’ = not developed, ‘Gran.’ = granulated, ‘Dif.’ = Differentiated or ‘Ripe’. ‘Ova max. dia’ refers to maximum diameter of ova. For nos. 134–157, only relatively low ovary mass were available (<2.5 kg) from which these were categorized as ‘immature’ or ‘mature’ in Aim 2 based on findings of Aim 1. ‘M. stage’ refers to maturity stage. Source refers to either J. Nielsen (JN), K. Yano (KY) or Bjørn Berland (BB) as the collector of data. (DOCX) [file pone.0238986.s009.docx]

**S2 Table (1 of 3)**.

| **No.** | **TL** | **Liver** | **Ovary** | | | **Uteri** | | **Ova** | | | **M. stage** | **Source** |
| --- | --- | --- | --- | --- | --- | --- | --- | --- | --- | --- | --- | --- |
|  | (m) | Mass (kg) | Mass (kg) | Length (cm) | Color | Size | Villi | Stage | Max. dia. (cm) | Count |  |  |
| 56 | 1.5 |  | <0.1 |  | White |  |  | Nd |  |  | 1 | JN |
| 57 | 1.6 | 2.5 | <0.1 |  | White | Small |  | Nd |  |  | 1 | JN |
| 58 | 2.7 | 13 | 0.2 |  | White |  |  | Nd |  |  | 1 | JN |
| 59 | 2.8 | 14 | 0.1 |  | White |  |  | Nd |  |  | 1 | JN |
| 60 | 2.8 | 23 | 0.2 |  |  |  |  | Nd |  |  | 1 | JN |
| 61 | 3.1 | 23 | 0.2 |  | White/red |  |  | Nd |  |  | 1 | JN |
| 62 | 3.1 | 34 | 0.5 |  | White |  |  | Nd |  |  | 1 | JN |
| 63 | 3.1 | 23 | 0.2 |  | White |  |  | Nd |  |  | 1 | JN |
| 64 | 3.1 | 40 |  |  | White |  |  | Nd |  |  | 1 | JN |
| 65 | 3.2 |  | 0.4 |  |  |  |  | Nd |  |  | 1 | JN |
| 66 | 3.2 |  |  |  | White |  |  | Nd |  |  | 1 | JN |
| 67 | 3.2 | 31 | 0.4 |  | White/red |  |  | Nd |  |  | 1 | JN |
| 68 | 3.2 | 27 |  |  | White |  |  | Nd |  |  | 1 | JN |
| 69 | 3.2 | 45 | 0.4 |  | White |  |  | Nd |  |  | 1 | JN |
| 70 | 3.2 |  |  | 50 | White |  |  | Nd |  |  | 1 | JN |
| 71 | 3.2 | 31 | 0.3 |  | White |  |  | Nd |  |  | 1 | JN |
| 72 | 3.2 | 31 | 0.3 |  | White |  |  | Nd |  |  | 1 | JN |
| 73 | 3.4 | 32 | 0.2 |  | White |  |  | Nd |  |  | 1 | JN |
| 74 | 3.5 | 47 | 0.5 |  | White/red |  |  | Nd |  |  | 1 | JN |
| 75 | 3.5 | 65 | 0.5 |  | White |  |  | Nd |  |  | 1 | JN |
| 76 | 3.8 | 56 | 1.0 | 46 | Reddish |  |  | Gran | 0.2 |  | 1 | JN |
| 77 | 3.8 | 91 | 0.7 |  |  |  |  | Nd |  |  | 1 | JN |
| 78 | 3.9 | 73 | 0.7 | 43 | White |  |  | Nd |  |  | 1 | JN |
| 79 | 3.9 | 53 | 0.9 |  | White/red |  |  | Gran |  |  | 1 | JN |
| 80 | 3.9 | 40 | 0.7 | 62 | White | Small | No | Gran |  |  | 1 | JN |
| 81 | 4.1 | 95 | 0.7 | 48 | White red |  |  | Nd |  |  | 1 | JN |
| 82 | 4.0 |  |  | 65 |  |  | No |  | 1.5 |  | 2 | BB |
| 83 | 4.0 |  |  | 100 |  |  | No |  | 3.5 |  | 2 | BB |
| 84 | 4.0 |  |  |  |  |  | No |  | 4.0 |  | 2 | BB |
| 85 | 4.0 |  |  |  |  |  | No |  | 4.5 |  | 2 | BB |
| 86 | 4.1 |  |  | 80 |  |  | No |  | 1.5 |  | 2 | BB |
| 87 | 4.1 |  |  | 80 |  |  | No |  | 2.5 |  | 2 | BB |
| 88 | 4.1 |  |  | 60 |  |  | No |  | 1.0 |  | 2 | BB |
| 89 | 4.2 |  |  | 80 |  |  | No |  | 1.0 |  | 2 | BB |
| 90 | 4.2 |  |  | 88 |  |  | No |  | 2.5 |  | 2 | BB |
| 91 | 4.2 |  |  |  |  |  | No |  | 1.3 |  | 2 | BB |

**S2 Table (2 of 3)**.

| **No.** | **TL** | **Liver** | **Ovary** | | | **Uteri** | | **Ova** | | | **M. stage** | **Source** |
| --- | --- | --- | --- | --- | --- | --- | --- | --- | --- | --- | --- | --- |
|  | (m) | Mass (kg) | Mass (kg) | Length (cm) | Color | Mass (kg) | Villi | Stage | Max. dia. (cm) | Count |  |  |
| 92 | 4.3 | 236 | 11.9 | 76 | White/red | Small | No | Dif | 3.5 | 1,327 | 2 | JN |
| 93 | 4.3 |  |  | 75 |  |  | No |  | 1.0 |  | 2 | BB |
| 94 | 4.3 |  |  |  |  |  | No |  | 3.5 |  | 2 | BB |
| 95 | 4.4 |  |  | 75 |  |  | No |  | 1.0 |  | 2 | BB |
| 96 | 4.5 |  |  | 85 |  |  | No |  | 3.5 |  | 2 | BB |
| 97 | 4.5 |  |  | 90 |  |  | No |  | 1.8 |  | 2 | BB |
| 98 | 4.5 |  |  | 90 |  |  | No |  | 3.0 |  | 2 | BB |
| 99 | 4.5 |  |  | 110 |  |  | No |  | 3.5 |  | 2 | BB |
| 100 | 4.5 |  |  | 115 |  |  | No |  | 4.0 |  | 2 | BB |
| 101 | 4.5 | 274 | 2.5 | 75 | White/red | Small | No | Dif | 1.5 |  | 2 | JN |
| 102 | 4.8 |  |  | 100 |  |  | No |  |  |  | 2 | BB |
| 103 | 4.0 |  |  | 80 |  |  | Yes |  |  |  | 2(7) | BB |
| 104 | 4.0 |  |  | 100 |  |  | Yes |  |  |  | 2(7) | BB |
| 105 | 4.1 |  |  | 85 |  |  | Yes |  | 1.0 |  | 2(7) | BB |
| 106 | 4.2 | 129 | 3.0 | 65 | White/red | Enlarged | Yes | Dif | 1.0 |  | 2(7) | JN |
| 107 | 4.2 |  |  | 70 |  |  | Yes |  | 1.0 |  | 2(7) | BB |
| 108 | 4.2 |  |  | 85 |  |  | Yes |  | 1.5 |  | 2(7) | BB |
| 109 | 4.2 |  |  | 75 |  |  | Yes |  |  |  | 2(7) | BB |
| 110 | 4.2 |  |  | 82 |  |  | Yes |  |  |  | 2(7) | BB |
| 111 | 4.3 |  |  | 80 |  |  | Yes |  | 1.3 |  | 2(7) | BB |
| 112 | 4.3 | 85 | 2.1 | 71 | White/red | Enlarged | Yes | Dif | 1.0 |  | 2(7) | JN |
| 113 | 4.3 |  |  | 65 |  |  | Yes |  |  |  | 2(7) | BB |
| 114 | 4.4 |  |  | 80 |  |  | Yes |  |  |  | 2(7) | BB |
| 115 | 4.4 | 100 | 2.7 | 81 | White | Enlarged | Yes | Dif | 1.1 |  | 2(7) | JN |
| 116 | 4.4 |  |  | 72 |  |  | Yes |  | 1.0 |  | 2(7) | BB |
| 117 | 4.5 |  |  | 100 |  |  | Yes |  | 1.5 |  | 2(7) | BB |
| 118 | 4.5 |  |  | 80 |  |  | Yes |  |  |  | 2(7) | BB |
| 119 | 4.7 | 57 | 2.2 | 68 | White/red | Enlarged | Yes | Dif | 1.0 |  | 2(7) | JN |
| 120 | 4.8 |  |  | 80 |  |  | Yes |  |  |  | 2(7) | BB |
| 121 | 4.9 |  |  | 100 |  |  | Yes |  | 1.3 |  | 2(7) | BB |
| 122 | 4.1 | 144 | 2.2 | 67 | White/red |  |  | Dif | 1.3 |  | 2(?) | JN |
| 123 | 4.3 | 178 | 4.5 | 81 | White/red |  |  | Dif | 2.2 |  | 2(?) | JN |
| 124 | 4.5 | 146 | 3.1 |  |  |  |  |  |  |  | 2(?) | JN |
| 125 | 4.5 | 160 | 2.1 | 69 | White/red |  |  | Dif | 1.0 |  | 2(?) | JN |
| 126 | 4.0 |  |  | 150 |  |  | No | Ripe | 6.0 |  | 3 | BB |
| 127 | 4.1 |  |  | 140 |  |  | Yes | Ripe | 6.0 |  | 3(7) | BB |
| 128† | 4.7 | 158 | 80.0 |  | Yellow | Enlarged | Yes | Ripe | 6.0 | 649 | 3(7) | JN |
| 129* | 4.2 |  |  | 180 |  |  |  | Ripe | 7.0 | ~400 | 3 (?) | BB |
| 130 | 4.1 |  |  | 120 |  |  | No |  | 5.0 |  | 3 | BB |
| † Body mass calculated according to girth (3.1 m) and length from Nielsen et al. 2014 to 1,367 kg. Mean diameter±SD and weight±SD for subsample of 25 ova was 5.5±0.2 mm and 115.6±10.83 gr, respectively.  * maximum ova diameter of 7.0 cm but also the size of majority of ova. | | | | | | | | | | | | |

**S2 Table (3 of 3).**

| **No.** | **TL** | **Liver** | **Ovary** | | | **Uteri** | | **Ova** | | | **M. stage** | **Source** |
| --- | --- | --- | --- | --- | --- | --- | --- | --- | --- | --- | --- | --- |
|  | (m) | Mass (kg) | Mass (kg) | Length (cm) | Color | Mass (kg) | Villi | Stage | Max. dia. (cm) | Count |  |  |
| 131 | 4.5 |  |  | 120 |  |  | No |  | 5.0 |  | 3 | BB |
| 132†† | 4.5 |  | 49.0 |  | Yellow |  |  | Ripe | 7.0 | 455 | 3(?) | JN |
| 133‡ | 4.3 | 116 | 4.2 | 70 | Brownish | Enlarged | Yes | Nd |  |  | 7 | JN |
| 134 | 0.7 |  | <0.1 |  |  |  |  |  |  |  | Cat. ‘Immature’ | KY |
| 135 | 2.1 |  | 0.2 |  |  |  |  |  |  |  | Cat. ‘Immature’ | KY |
| 136 | 2.3 |  | 0.1 |  |  |  |  |  |  |  | Cat. ‘Immature’ | KY |
| 137 | 2.7 |  | 0.2 |  |  |  |  |  |  |  | Cat. ‘Immature’ | KY |
| 138 | 2.9 |  | 0.3 |  |  |  |  |  |  |  | Cat. ‘Immature’ | KY |
| 139 | 2.9 |  | 0.2 |  |  |  |  |  |  |  | Cat. ‘Immature’ | KY |
| 140 | 3.1 |  | 0.5 |  |  |  |  |  |  |  | Cat. ‘Immature’ | KY |
| 141 | 3.3 |  | 0.5 |  |  |  |  |  |  |  | Cat. ‘Immature’ | KY |
| 142 | 3.6 |  | 0.7 |  |  |  |  |  |  |  | Cat. ‘Immature’ | KY |
| 143 | 3.7 |  | 0.9 |  |  |  |  |  |  |  | Cat. ‘Immature’ | KY |
| 144 | 3.8 |  | 0.5 |  |  |  |  |  |  |  | Cat. ‘Immature’ | KY |
| 145 | 3.8 |  | 0.8 |  |  |  |  |  |  |  | Cat. ‘Immature’ | KY |
| 146 | 4.0 |  | 0.6 |  |  |  |  |  |  |  | Cat. ‘Immature’ | KY |
| 147 | 4.0 |  | 0.8 |  |  |  |  |  |  |  | Cat. ‘Immature’ | KY |
| 148 | 4.0 |  | 1.4 |  |  |  |  |  |  |  | Cat. ‘Immature’ | KY |
| 149 | 4.1 |  | 0.8 |  |  |  |  |  |  |  | Cat. ‘Immature’ | KY |
| 150 | 4.3 |  | 1.1 |  |  |  |  |  |  |  | Cat. ‘Immature’ | KY |
| 151 | 4.4 |  | 1.2 |  |  |  |  |  |  |  | Cat. ‘Immature’ | KY |
| 152 | 4.4 |  | 1.2 |  |  |  |  |  |  |  | Cat. ‘Immature’ | KY |
| 153 | 4.4 |  | 1.4 |  |  |  |  |  |  |  | Cat. ‘Immature’ | KY |
| 154 | 4.5 |  | 0.8 |  |  |  |  |  |  |  | Cat. ‘Immature’ | KY |
| 155 | 4.6 |  | 1.0 |  |  |  |  |  |  |  | Cat. ‘Immature’ | KY |
| 156 | 4.8 |  | 1.7 |  |  |  |  |  |  |  | Cat. ‘Immature’ | KY |
| 157 | 4.5 |  | 2.5 |  |  |  |  |  |  |  | Cat. ‘Mature’ | KY |
| ††Body mass was measured to 1,100 kg on electronic scale. For a subsample of 25 ova mean diameter±SD is 5.1±0.6 cm  ‡ Ovaries seems exhausted and uteri very extended as if they are in a resting phase after a recent pregnancy (see Fig 5b) | | | | | | | | | | | | |
