# Supplementary material for: Assessing the reproductive biology of the Greenland shark (Somniosus microcephalus)
Source: PLoS One. 2020 Oct 7;15(10):e0238986. doi: 10.1371/journal.pone.0238986 (PMC7540863; doi:10.1371/journal.pone.0238986)
Supplement: S3 Table — Female sharks categorized on sight as either ‘Immature’, ‘Maturing’ or ‘Mature’ by Berland. Shark identification numbers are continuous from previous S1 and S2 Tables. (DOCX) [file pone.0238986.s010.docx]

**S3 Table**

| **No.** | **TL** | **Stage** | **No.** | **TL** | **Stage** | **No.** | **TL** | **Stage** | **No.** | **TL** | **Stage** |
| --- | --- | --- | --- | --- | --- | --- | --- | --- | --- | --- | --- |
|  | (m) |  |  | (m) |  |  | (m) |  |  | (m) |  |
| 158 | 3.4 | Immature | 199 | 4.0 | Immature | 240 | 4.2 | Immature | 281 | 4.1 | Mature |
| 159 | 3.5 | Immature | 200 | 4.0 | Immature | 241 | 4.2 | Immature | 282 | 4.1 | Mature |
| 160 | 3.5 | Immature | 201 | 4.0 | Immature | 242 | 4.2 | Immature | 283 | 4.2 | Mature |
| 161 | 3.5 | Immature | 202 | 4.0 | Immature | 243 | 4.2 | Immature | 284 | 4.2 | Mature |
| 162 | 3.5 | Immature | 203 | 4.0 | Immature | 244 | 4.3 | Immature | 285 | 4.2 | Mature |
| 163 | 3.5 | Immature | 204 | 4.0 | Immature | 245 | 4.3 | Immature | 286 | 4.2 | Mature |
| 164 | 3.5 | Immature | 205 | 4.0 | Immature | 246 | 4.3 | Immature | 287 | 4.2 | Mature |
| 165 | 3.6 | Immature | 206 | 4.0 | Immature | 247 | 4.3 | Immature | 288 | 4.2 | Mature |
| 166 | 3.6 | Immature | 207 | 4.0 | Immature | 248 | 4.4 | Immature | 289 | 4.2 | Mature |
| 167 | 3.6 | Immature | 208 | 4.0 | Immature | 249 | 4.4 | Immature | 290 | 4.3 | Mature |
| 168 | 3.6 | Immature | 209 | 4.0 | Immature | 250 | 4.4 | Immature | 291 | 4.3 | Mature |
| 169 | 3.6 | Immature | 210 | 4.0 | Immature | 251 | 4.4 | Immature | 292 | 4.3 | Mature |
| 170 | 3.6 | Immature | 211 | 4.0 | Immature | 252 | 4.4 | Immature | 293 | 4.3 | Mature |
| 171 | 3.7 | Immature | 212 | 4.0 | Immature | 253 | 4.4 | Immature | 294 | 4.3 | Mature |
| 172 | 3.7 | Immature | 213 | 4.0 | Immature | 254 | 4.5 | Immature | 295 | 4.3 | Mature |
| 173 | 3.7 | Immature | 214 | 4.0 | Immature | 255 | 4.5 | Immature | 296 | 4.3 | Mature |
| 174 | 3.7 | Immature | 215 | 4.0 | Immature | 256 | 4.5 | Immature | 297 | 4.3 | Mature |
| 175 | 3.7 | Immature | 216 | 4.0 | Immature | 257 | 4.5 | Immature | 298 | 4.3 | Mature |
| 176 | 3.7 | Immature | 217 | 4.0 | Immature | 258 | 4.7 | Immature | 299 | 4.4 | Mature |
| 177 | 3.7 | Immature | 218 | 4.0 | Immature | 259 | 4.7 | Immature | 300 | 4.4 | Mature |
| 178 | 3.7 | Immature | 219 | 4.0 | Immature | 260 | 3.9 | Maturing | 301 | 4.4 | Mature |
| 179 | 3.7 | Immature | 220 | 4.0 | Immature | 261 | 3.9 | Maturing | 302 | 4.4 | Mature |
| 180 | 3.7 | Immature | 221 | 4.1 | Immature | 262 | 4.0 | Maturing | 303 | 4.4 | Mature |
| 181 | 3.8 | Immature | 222 | 4.1 | Immature | 263 | 4.0 | Maturing | 304 | 4.5 | Mature |
| 182 | 3.8 | Immature | 223 | 4.1 | Immature | 264 | 4.1 | Maturing | 305 | 4.5 | Mature |
| 183 | 3.8 | Immature | 224 | 4.1 | Immature | 265 | 4.1 | Maturing | 306 | 4.5 | Mature |
| 184 | 3.8 | Immature | 225 | 4.1 | Immature | 266 | 4.2 | Maturing | 307 | 4.5 | Mature |
| 185 | 3.8 | Immature | 226 | 4.1 | Immature | 267 | 4.2 | Maturing | 308 | 4.5 | Mature |
| 186 | 3.8 | Immature | 227 | 4.1 | Immature | 268 | 4.4 | Maturing | 309 | 4.5 | Mature |
| 187 | 3.8 | Immature | 228 | 4.1 | Immature | 269 | 4.4 | Maturing | 310 | 4.6 | Mature |
| 188 | 3.8 | Immature | 229 | 4.1 | Immature | 270 | 4.4 | Maturing | 311 | 4.8 | Mature |
| 189 | 3.8 | Immature | 230 | 4.1 | Immature | 271 | 4.8 | Maturing | 312 | 4.9 | Mature |
| 190 | 3.8 | Immature | 231 | 4.1 | Immature | 272 | 4.0 | Mature |  |  |  |
| 191 | 3.8 | Immature | 232 | 4.1 | Immature | 273 | 4.0 | Mature |  |  |  |
| 192 | 3.9 | Immature | 233 | 4.1 | Immature | 274 | 4.0 | Mature |  |  |  |
| 193 | 3.9 | Immature | 234 | 4.2 | Immature | 275 | 4.0 | Mature |  |  |  |
| 194 | 3.9 | Immature | 235 | 4.2 | Immature | 276 | 4.0 | Mature |  |  |  |
| 195 | 3.9 | Immature | 236 | 4.2 | Immature | 277 | 4.1 | Mature |  |  |  |
| 196 | 3.9 | Immature | 237 | 4.2 | Immature | 278 | 4.1 | Mature |  |  |  |
| 197 | 3.9 | immature | 238 | 4.2 | Immature | 279 | 4.1 | Mature |  |  |  |
| 198 | 4.0 | Immature | 239 | 4.2 | Immature | 280 | 4.1 | Mature |  |  |  |
