# Supplementary material for: Assessing the reproductive biology of the Greenland shark (Somniosus microcephalus)
Source: PLoS One. 2020 Oct 7;15(10):e0238986. doi: 10.1371/journal.pone.0238986 (PMC7540863; doi:10.1371/journal.pone.0238986)
Supplement: S4 Table — Reproductive strategy (Repr. strat.) can either be Lec = Lecitotrophic, Ma = Matrotrophic or UA = Unassessed. Mean values are in []. Ovarian cycle is either concurrent (conc.) or non-concurrent (N-conc.). Abbreviations for regions are: Atl. = Atlantic Ocean, Med. = Mediterranean, Pac. = Pacific, Indi. = Indian Ocean, Arab. = Arabian Sea, Bl. Sea = Black Sea. (DOCX) [file pone.0238986.s011.docx]

**S4 Table (1 of 4)**

|  | **Region** | **N_females_**  (N_mature_ ) | **Fec_ovarian_**  range (mean) | **Fec_uterine_**  range (mean) | **Ripe ova dia.**  cm (mean) | **Birth size,** TL  cm (mean) | **Correlation Fec+Size_mat._** | **Ovarian cycle** | **Repr.**  **strat.** |
| --- | --- | --- | --- | --- | --- | --- | --- | --- | --- |
| **Centrophoridae – Gulper sharks** | | |  |  |  |  |  |  |  |
| *Centrophorus squamosus,* TL_max_~1.6 m | | |  |  |  |  |  |  |  |
| Girard & Du Buit 1999 [30] | NE Atl. | 417 (53) | 7-11 (10.0) |  | 7.0-8.5 |  |  |  |  |
| Clarke et al. 2001 [31] | NE Atl. | 431 (83) | 6-11 (8.1) |  |  |  |  |  |  |
| Bañón et al. 2006 [33] | NE Atl. | 318 | 7-12 (9) | 7-7 [N=2] |  | 38-40 |  |  |  |
| Figueiredo et al. 2008 [35] | NE Atl. | 467 | 5-15 | 6 [N=1] |  | (44.0) | Yes | Conc. |  |
| Severino et al. 2009 [36] | NE Atl. | 61 |  | 2-10 (5.4) |  |  |  |  |  |
| *Centrophorus granulosus*, TL_max_~1.8 m | | |  |  |  |  |  |  |  |
| Cotton et al. 2015 [23] | NW Atl. | 118 |  | 4-7 (5.3) | Up to 9.0 | 37.4-39.2 | No | Conc. | Lec |
| Banón et al. 2008 [34] | NE Atl. | 156 | 1-10 (5) | 1-6 (3) | 5.5-8.0 (6.9) | 35-47 (40) |  | Conc. |  |
| *Centrophorus uyato*, TL_max_~1.0 m | | | | | | | | |  |
| Guallart & Vicent 2001* [21] | Med. |  |  |  | 6.3-8.7 | 28.8-35.7 |  |  | Lec |
| *Centrophorus harrisoni*, TL_max_~0.8 m | | |  |  |  |  |  |  |  |
| Graham & Daley 2011 [29] | SW Pac. | 86 (41) | 1-2 | 1-2 | Up to 8.2 | 35-40 |  | Conc. |  |
| *Centrophorus moluccensis*, TL_max_~1.0 m | | |  |  |  |  |  |  |  |
| Graham & Daley 2011 [29] | SW Pac. | 68 (32) | 1-2 | 1-2 | 4.5-6.5 | 34-35 |  | Conc. |  |
| *Centrophorus* sp*.* 1 | | | | | | | | |  |
| Graham & Daley 2011** [29] | SW Pac. | 100 (56) | 1 | 1 | 8.0-8.5 | 38-45 |  | Conc. |  |
| *Centrophorus* sp. 2 | | | | |  |  |  |  |  |
| McLaughlin & Morissey 2005*** [32] | W Atl. | 51 (41) | 1-3 | 1-2 |  | 34-35 |  | Conc. |  |
| *Deania profundorum*, TL_max_~0.8 m | | |  |  |  |  |  |  |  |
| Sousa et al. 2009 [37] | N Atl. | 351 | 6-14 | 6-11 | 1.6-5.2 | 27 |  | Conc. |  |
|  |  |  |  |  |  |  |  |  |  |
| **Dalatiidae – Kitefin sharks** |  |  |  |  |  |  |  |  |  |
| *Dalatias licha*, TL_max_~1.6 m |  |  |  |  |  |  |  |  |  |
| Silva 1988 [39] | M Atl. | 67 | 10-18 | 10-14 |  | ~42 |  |  |  |
| Capapé et al. 2008 [40] | Med. | 10 |  | 6 [N=1] |  | 32-39 |  |  |  |
| Bass et al. 1976 [38] |  |  | 10-20 |  | 7.0-9.0 |  |  | N.-conc. |  |
|  |  |  |  |  |  |  |  |  |  |
| **Echinorhinidae – Bramble sharks** | |  |  |  |  |  |  |  |  |
| *Echinorhinus brucus,* TL_max_~3.2 m |  |  |  |  |  |  |  |  |  |
| Joel & Ebenzer 1991 [41] | Indi. | 1 |  | 52 |  |  |  |  |  |
| Akhilesh et al. 2013 [42] | Arab. | 256 | 12-38 | 10-36 | Up to 8.0 | 42-46 |  |  |  |
|  |  |  |  |  |  |  |  |  |  |
| * Birth size in precaudal length (not TL). Reported as *C. granolusus* but suggested *C. uyato* according to Veríssimo et al. 2014 [53]  ** Reported as *Centrophorus zeehaani* but suggested *C. uyato* according to Veríssimo et al. 2014 [52]. Listed here as sp. 1 due to capture location in the Pacific which is different from *C. uyato* in the Mediterranean by Guallart & Vicent 2001 [22].  *** Reported as *C.* cf. *uyato* but re-described as a clade B by Veríssimo et al. 2014 [53]. | | | | | | | | | |

**S4 Table (2 of 4)**

|  | **Region** | **N_females_**  (mature) | **Fec_ovarian_**  range (mean) | **Fec_uterine_**  range (mean) | **Ripe ova dia.**  cm (mean) | **Birth size,** TL  cm (mean) | **Correlation Fec+Size_mat._** | **Ovarian cycle** | **Repr.**  **strat.** |
| --- | --- | --- | --- | --- | --- | --- | --- | --- | --- |
| **Oxynotidae – Rough sharks** |  |  |  |  |  |  |  |  |  |
| *Oxynotus bruniensis,* TL_max_~0.7 m |  |  |  |  |  |  |  |  |  |
| Finucci et al. 2016 [46] | SW Pac. | 45 | 8-17 (11.1) | 8 (8) [N=3] | Up to 5.1 | 25-27 |  | N-conc. |  |
| *Oxynotus centrina,* TL_max_~1.5 m |  |  |  |  |  |  |  |  |  |
| Capapé et al. 1999 [43] | Medi. | 49 (19) | 9-22 (14.5) | 10-12 (10.7) | 3.8-4.1 (3.9) | 21-24 (22.1) |  | N-conc. |  |
| Megalofonou & Damalas 2004 [44] | Medi. | 1 |  | 15 |  |  |  |  |  |
| Basusta et al. 2005 [45] | Medi. | 1 | 11 |  |  |  |  |  |  |
|  |  |  |  |  |  |  |  |  |  |
| **Etmopteridae – Lantern sharks** |  |  |  |  |  |  |  |  |  |
| *Centroscyllium fabricii*, TL_max_~0.8 m |  |  |  |  |  |  |  |  |  |
| Yano 1995*** [27] | NW Atl. | 1476 | 5-47 | 4-35 | 3.0-3.5 | 15.2-19.2 | Yes | N-conc. |  |
| Hedeholm et al. 2019 [50] | NW Atl. | 158 |  | 14-32 |  | ~16 |  |  |  |
| J. Nielsen unpublished data | NW Atl. | 56 | 19-41 | 15 |  | 15.3-16.1 |  |  |  |
| *Etmopterus princeps*, TL_max_~0.8 m | |  |  |  |  |  |  |  |  |
| Cotton et al. 2015 [23] | M Atl. | 269 |  | 7-18 (11.2) |  | 15.7-17.5 | No | N-conc. | Mat |
| *Etmopterus granulosus*, TL_max_~0.8 m | | |  |  |  |  |  |  |  |
| Wetherbee 1996 [47] | SW Paci. | 492 | 7-30 (14.9) | 9-15 (12.7) | 4.0-4.5 |  |  | N-conc. |  |
| *Etmopterus spinax,* TL_max_~0.6 m | | |  |  |  |  |  |  |  |
| Capapé et al. 2001 [48] | Med. | 179 | 9-17 (13.5) | 5-9 (6.8) | 2.4-2.7 | 11.9-13.3 (12.6) | Yes | N-conc. |  |
| Coelho & Erzini 2008 [49] | NE Atl. | 485 | 5-21 (9.9) | 1-16 (7.6) |  |  | Yes | N-conc. |  |
|  |  |  |  |  |  |  |  |  |  |
| **Somniosidae – Sleeper sharks** |  |  |  |  |  |  |  |  |  |
| *Centroscymnus coelolepis*, TL_max_~1.1 m |  |  |  |  |  |  |  |  |  |
| Yano & Tanaka 1988 [51] | W Pac. | 44 | 23, 24 | 15-29 (22.0) | 4.0-5.5 |  | Yes | N-conc. |  |
| Girard & Du Buit 1999 [30] | NE Atl. | (122) | 8-22 (16) | 8-19 (14.0) | ~6.0 | ~30 | Yes |  |  |
| Clarke et al. 2001 [31] | NE Atl. | 388 (281) | 10-21 (12.7) | 8-21 (13.8) | 4.5-6.5 |  |  | N-conc. |  |
| Veríssimo et al. 2003 [52] | NE Atl. | 871 | 5-30 (13.2) | 1-25 (9.9) | 4.5-8.8 (6.5) | 23-30 (27) | No | N-conc. |  |
| Bañon et al. 2006 [33] | NE Atl. | 317 | 23 (N=1) | 5-22 (14) |  | 27-29 |  |  |  |
| Figueiredo et al. 2008 [35] | NE Atl. | 1773 | (13.7) | (11.3) |  | ~31 | No | N-conc. |  |
| Moura et al. 2011 [22] | NE Atl. |  |  |  |  |  |  |  | Lec |
| *Centroscymnus owstoni*, TL_max_~1.0 m |  |  |  |  |  |  |  |  |  |
| Yano & Tanaka 1988 [51] | W Pac | 316 | 20-28 (25.7) | 16-28 (22.1) | 5.0-6.0 |  | Yes | N-conc. |  |
| *** Numbers on ovarian and uterine fecundity extracted from Fig. 9 in Yano 1995 and not from the text. | | | | | | | | | |

**S4 Table (3 of 4)**

|  | **Region** | **N_females_**  (mature) | **Fec_ovarian_**  range (mean) | **Fec_uterine_**  range (mean) | **Ripe ova dia.**  cm (mean) | **Birth size,** TL  cm (mean) | **Correlation Fec+Size_mat._** | **Ovarian cycle** | **Repr.**  **strat.** |
| --- | --- | --- | --- | --- | --- | --- | --- | --- | --- |
| *Scymnodalatias albicauda,* TL_max_~1.1 m | | |  |  |  |  |  |  |  |
| Nakaya & Nakano 1995 [53] | S Atl. | 1 |  | 59 |  |  |  |  |  |
| *Somniosus rostratus*, TL_max_~1.4 m | |  |  |  |  |  |  |  |  |
| Guallart & Nielsen unpublished data | Medi. | (27) | 23 | 6-21 | 5.2-5.7 (5.5) | 21-28 | No |  |  |
|  |  |  |  |  |  |  |  |  |  |
| **Squalidae – Dogfish sharks** |  |  |  |  |  |  |  |  |  |
| *Squalus acanthias,* TL_max_~1.2 m |  |  |  |  |  |  |  |  |  |
| Ranzi 1934 [54] | NA | NA |  |  |  |  |  |  | Lec. |
| Nammack et al. 1985 [57] | NW Atl. | (491) | 1-18 (7.9) | 2-15 (6.6) | 4.0-4.8 | 23-29 | Yes | Conc. |  |
| Hanchet 1988 [58] | SW Pac. | 2567 |  | 1-16 (5.1) | (4.2) | 18-30 (24) | Yes + pup size |  |  |
| Avsar 2001 [59] | Bl. Sea | 160 (90) |  | 2-17 (8.2) |  |  | Yes |  |  |
| Jones & Ugland 2001 [63] | NW Atl. | 233 | 3-17 (8.9) | 2-15 |  | Up to 24 | Yes |  |  |
| Henderson et al. 2002 [60] | NE Atl. | 132 | 4-13 [7.7] | 4-16 |  |  | No |  |  |
| *Squalus megalops,* TL_max_~0.7 m | |  |  |  |  |  |  |  |  |
| Watson & Smale 1998 [64] | SW Indi. | (1546) | 2-4 | 2-4 | ~4.0-4.2 | 23.2-27.7 | Yes+pup size | Conc. |  |
| Braccini et al. 2006 [61] | SW Pac. | 722 |  | 2-4 | ~4.0-4.9 | 19.1.24.4 | Yes+pup size | Conc. |  |
| Braccini et al. 2007 [62] | SW Pac |  |  | 2-4 |  | 18.0-24.4 | Pup size |  | Lec |
| *Squalus suckleyi*, TL_max_~1.6 m |  |  |  |  |  |  |  |  |  |
| Ketchen 1972 [55] | NE Pac. | (630) | 2-13 [6.8] | 2-16 (6.6) | 3.0-4.0 | (27) | Yes |  |  |
| Jones & Geen 1977**** [56] | NE Pac. | 500 |  | 4-11 (7.1) | ~4.0 | (26) |  |  |  |
|  |  |  |  |  |  |  |  |  |  |
| **** Originally described as *S. acanthias*. | | | | | | | | | |
|  | | | | | | | | | |
